# Dynamic Stability and Trunk Control Improvements Following Robotic Balance and Core Stability Training in Chronic Stroke Survivors: A Pilot Study

**DOI:** 10.3389/fneur.2020.00494

**Published:** 2020-06-17

**Authors:** Alice De Luca, Valentina Squeri, Laura M. Barone, Honorè Vernetti Mansin, Serena Ricci, Ivano Pisu, Cinzia Cassiano, Cristina Capra, Carmelo Lentino, Lorenzo De Michieli, Carlo A. Sanfilippo, Jody A. Saglia, Giovanni A. Checchia

**Affiliations:** ^1^Movendo Technology, Genoa, Italy; ^2^Recovery and Functional Reeducation Unit, Santa Corona Hospital, ASL2 Savonese, Pietra Ligure, Italy; ^3^Department of Informatics, Bioengineering, Robotics, and System Engineering, University of Genoa, Genoa, Italy; ^4^Rehab Technologies, IIT, Genoa, Italy; ^5^Department of Rehabilitation, Local Health Agency EUGANEA, Padua, Italy

**Keywords:** stroke, robotic rehabilitation, balance, core stability, trunk control

## Abstract

Stroke survivors show greater postural oscillations and altered muscular activation compared to healthy controls. This results in difficulties in walking and standing, and in an increased risk of falls. A proper control of the trunk is related to a stable walk and to a lower falling risk; to this extent, rehabilitative protocols are currently working on core stability. The main objective of this work was to evaluate the effectiveness of trunk and balance training performed with a new robotic device designed for evaluation and training of balance and core stability, in improving the recovery of chronic stroke patients compared with a traditional physical therapy program. Thirty chronic stroke patients, randomly divided in two groups, either underwent a traditional rehabilitative protocol, or a robot-based program. Each patient was assessed before and after the rehabilitation and at 3-months follow-up with clinical and robot-based evaluation exercises focused on static and dynamic balance and trunk control. Results from clinical scores showed an improvement in both groups in balance and trunk control. Robot-based indices analysis indicated that the experimental group showed greater improvements in proprioceptive control, reactive balance and postural control in unstable conditions, compared to the control group, showing an improved trunk control with reduced compensatory strategies at the end of the training. Moreover, the experimental group had an increased retention of the benefits obtained with training at 3 months follow up. These results support the idea that such robotic device is a promising tool for stroke rehabilitation.

## Introduction

Stroke is a cerebrovascular disease affecting fifteen million people every year (1, 2). Among them, nearly 50% of the patients experiences long term disability and postural deficits caused by an asymmetric posture and difficulties in transferring load to the lower limbs (1). Additionally, stroke survivors show greater postural oscillations (3) and altered muscular activation (4) compared to healthy controls. For these reasons, they may have difficulties executing complex tasks and exhibit an increased risk of falling due to difficulties in walking and standing (5). In particular, the risk of falling increases toward the paretic side causing a limitation in functional abilities (6, 7). Chronic stroke patients typically experience improvements in mobility (8–10) and functional balance (11), but it is unclear whether this is related to specific exercise programs (12).

According to Shumway-Cook and Woollacott, balance can be subdivided into three components: static/dynamic steady state (i.e., maintaining a steady position in sitting, standing and walking), proactive (i.e., anticipation of a predicted disturbance), and reactive (i.e., compensation of a disturbance) balance (13). Since these components are equally important for balance management, it is crucial that balance treatments focus on all these components (14, 15).

Balance and walking impairments in stroke survivors can be caused by insufficient strength in trunk muscles (16–22). Indeed, sitting ability and trunk control are useful prognostic indicators of outcomes after stroke (23).

Sitting balance in stroke survivors is characterized by a poor antigravity response and a posterior-version of the pelvis (24). This results in a decreased displacement of the Center of Pressure (CoP) during trunk movements, with a greater involvement of the upper part of the trunk and a reduced anterior tilt of the pelvis, with generation of compensatory strategies.

Position sense is a component of proprioception and an essential element of postural control (25, 26). Stroke survivors present an impaired trunk position sense that may cause trunk instability (25); for this reason, training postural proprioception could be a potential intervention strategy for improving balance.

Core stability has been defined as the ability to stabilize the spine as a result of local muscle activity (27). The “core muscles” include many muscles supporting the lumbo-pelvic-hip complex. Thus, “core stability training” works on the activation of deep trunk muscles through selective pelvis movement and abdominal contractions (28, 29). In the last years, the concept of “core” and the need to retrain “core stability” have been a focus in low back pain rehabilitation and sports (30). Recently, several studies investigated the effect of a core stability training in stroke rehabilitation (28, 31).

There is strong evidence showing that trunk control and core stability training are able to improve sitting, standing balance, mobility, trunk control and neuromuscular integration (32–34), having positive effects on daily life activities (28, 31, 35).

Thus, rehabilitative protocols are currently working on core stability through abdominal, pelvic and lumbar muscles reinforcement (25).

Different factors can affect neuroplasticity and learning in stroke survivors. Therapy needs to be intensive, active and challenging for optimal recovery (36). In other words, the difficulty of each exercise should be adapted to patient's impairment, making sure all the patients perform challenging exercises that can enhance short and long term neuronal changes (37).

In this context, robotic systems for rehabilitation can be promising tools to personalize rehabilitative programs for stroke patients, providing intense and repeatable activities, measuring a subject's performance and giving the possibility to set exercise difficulty according to their residual abilities (36). In the present study, we used a robotic device designed for training and evaluation of core stability and balance. Such device allows the implementation of different dynamic environments that stimulate postural responses.

The main objective of this work was to evaluate the effectiveness of a robot-based trunk and balance training in improving the recovery in chronic stroke patients compared to a traditional physical therapy program. Both programs were focused on static and dynamic postural stability exercises in sitting and standing positions (38–45), postural symmetry in sitting and standing tasks (46, 47), and anticipatory postural adjustments training (48–51). Our hypothesis was that, by providing an intense, personalized and more challenging training, the robot-aided therapy could lead to better outcomes in balance and trunk control.

## Materials and Methods

### Subjects

Thirty patients matching the following inclusion criteria were enrolled in the study (Table 1):

- Age between 18 and 75 years;
- Unilateral stroke detected by magnetic resonance;
- Chronic stroke (more than 6 months after the disease onset);
- Berg Balance Scale (52) (BBS) ≥41/56;
- Ability to walk for at least ten meters;
- Intact cognitive status [Mini-mental State Examination (MMSE (53) >26/30 (54, 55) or Token Test (56) >26 (57) for patients with aphasia].

**Table 1:** Subjects data.

**Characteristics**	**Groups**		***p***
	**E**	**C**	
Gender	9 F (60%)	5 F (33.3%)	0.27
	6 M (40%)	10 M (66.7%)	
Age [y]	58.53 ± 1.87	63.46 ± 2.51	0.07
Etiology	I:10	I:11	1
	H:5	H:4	
Side	R: 7 (46.6%)	R: 9 (60%)	0.71
	L: 8 (53.4%)	L: 6 (40%)	
BBS [/56]	48.14 ± 0.27	49.30 ± 0.39	0.31
MBT [/28]	16.14 ± 0.33	16.15 ± 0.48	0.73
TIS [/23]	12.57 ± 0.13	12.23 ± 0.21	0.78

*E, experimental, C, control; Age is defined in years [y]; I, Ischemic; H, hemorrhagic; Side stands for affected side; L, left, R, right; BBS, Berg Balance Scale; MBT, Mini-BESTest; TIS, Trunk Impairment Scale. Values are expressed as mean ± SE (standard error) or number (%). The comparison of medians was performed with a Mann Whitney U Test, while Fisher's exact test was used for categorical data*.

In addition, subjects with visual, vestibular, orthopedic or other neurological diseases were excluded from the study.

Since this is the first explorative study investigating the use of this type of robotic device in a population of stroke survivors, we restricted the inclusion criteria and decided to consider only high functioning chronic stroke survivors, who can be more stable. This choice was made to have sufficient statistical power to document significant difference in a heterogeneous population; further, it ensured that improvements were due to the specific treatment and did not depend on spontaneous recovery.

Participants were enrolled among the outpatient population of Recovery and Functional Re-education Unit of the Santa Corona Hospital (Pietra Ligure, Savona, Italy). The regional ethical committee approved this study (CER Liguria register number: 340REG2015) and subjects gave informed consent conforming to the ethical standards of the 1964 Declaration of Helsinki.

### Experimental Protocol

After recruitment, subjects were randomly assigned to two groups: the experimental group (*N* = 15, age mean 58.53 ± 1.87 SE years, 9 females, 8 left side affected) which underwent a robot-based rehabilitative protocol and the control group (*N* = 15, age mean 63.46 ± 2.51 SE years, 5 females, 6 left side affected) which performed traditional rehabilitative sessions with physical therapists. Each patient was assessed before (T0) and after (T1) the rehabilitative treatment and at 3-months follow-up (T2).

**Figure 1:**
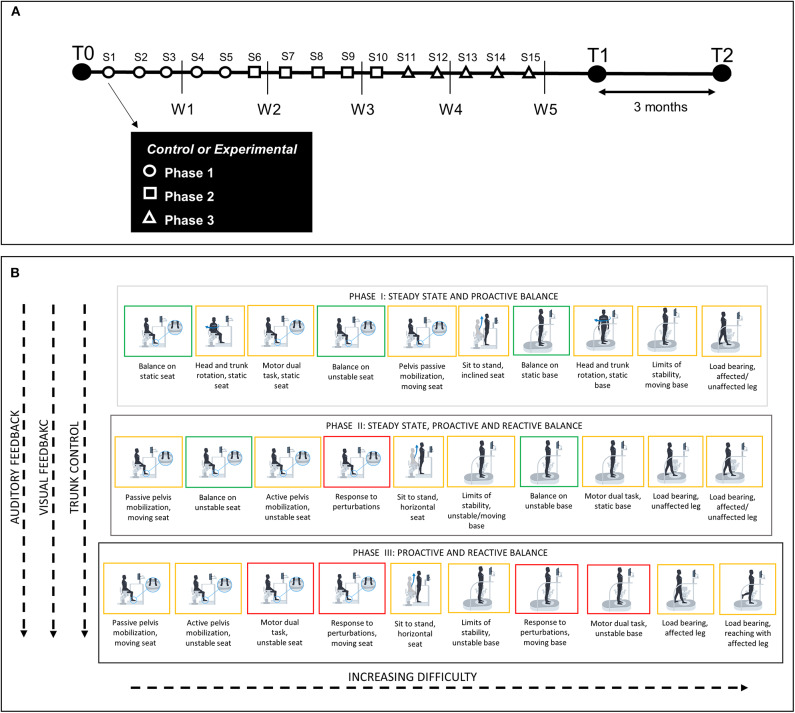
**(A)** Timeline of the experimental procedure. W, week of training; S, training session; T, time of evaluation; T0, before training; T1, after training; T2, 3 months after the end of the training. Each evaluation included clinical assessment and robot-based assessment. Experiment started with the first evaluation (T0), followed by 5 weeks of traditional (control) or robot-based (experimental) rehabilitation. Each week consisted of three sessions of exercises among three categories (steady state, proactive balance or reactive balance). Phase 1 (S1–S5) included steady state and proactive balance exercises; Phase 2 (S6–S10) included steady state, proactive and reactive balance exercises; Phase 3 (S11–S15) included proactive and reactive balance exercises. After training subjects were tested at the end of the program (T1) and at 3 months follow up (T2). **(B)** Summary of training activities on hunova for the experimental group. Each phase was characterized by different types of exercises that were presented with increasing difficulty. Auditory and visual feedbacks about the accuracy of the performance were continuously provided during the execution of the exercises. Gray-black blocks, different training phases; green blocks, steady state activities; orange blocks, proactive balance activities; red blocks, reactive balance activities.

After the first evaluation (T0), subjects started the rehabilitative program which lasted 5 weeks with three 45-min sessions per week (see the timeline of the experimental procedure in Figure 1A).

### Robotic Device

**Figure 2:**
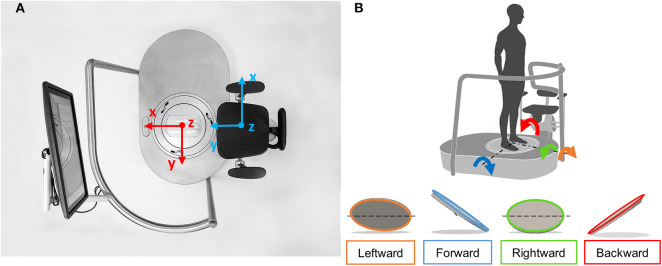
**(A)** Prototype of hunova: top view of the platform and seat. The screen was used in both the standing and sitting trials to give visual feedback of the subject's performance. References axis for the feet platform (in red) and seat platform (light blue) are represented. x indicates mediolateral direction, y indicates anteroposterior direction, z indicates vertical direction. **(B)** Reference system and platform inclinations in different directions (forward, backward, leftward, and rightward).

hunova (Movendo Technology srl, Genoa, IT) (58, 59) is a medical robotic device for the functional sensory-motor evaluation and rehabilitation of different body districts (from lower limbs to trunk) and functional abilities (balance and core stability) (Figure 2A). The device consists of two servo-controlled platforms having two degrees of freedom each one; one platform is positioned under the feet and the other is placed under the seat. This configuration allows to use the device both in standing—with double legs stance load, asymmetric double stance leg load or one leg stance load—and sitting positions (see Table S1 and Figure 1B for more details about configurations and patient's position on the device).

A six axis force-torque sensor is positioned under each platform, to measure the interaction forces and torques between the subject and the platforms, while optical incremental encoders measure the inclination of the platforms on the two axis (y axis—anteroposterior direction and x axis—medio lateral direction, see Figure 2A).

The robotic device is associated with a wireless inertial sensor (Inertial Movement Unit—IMU) placed on the patient's trunk, at the level of the sternum, allowing recording of the subject's trunk movements. The sensor includes an accelerometer, a gyroscope and a magnetometer. Thus, it measures the rotations along the x-axes (Roll) and y-axes (Pitch) and evaluates the movements of the patient's trunk in the frontal and sagittal plane, as well as movement accelerations. The platforms can be static or can operate in “passive” and “active” modes. In the passive mode, the movement of the platform does not depend on the subject, but the system controls speed and interaction (force and torque), according to a pre-programmed rotation imposed by the device motors. In the active mode, the subject controls the movement of the platform/seat with feet/pelvis movements, respectively, while the platform/seat exerts a certain resistance proportional to the subject's movement. Different kind of resistances, each one requiring different effort by the subject, can be set:

- “elastic” resistance, which simulates an elastic dynamic (i.e., an elastic rotatory force filed that tend to restore the platform position parallel to the floor with a force that is proportional to the platform displacement);
- “viscous” resistance, which simulates the dynamic of a fluid (the interaction force is proportional to the displacement velocity);
- no resistance or “proprioceptive” mode, in which the platforms are completely free, and do not exert any type of resistance.

Various configurations (subject seated or standing) targeting different body regions (from lower limbs to trunk), allow for a large amount of exercises to be performed. In addition, the possibility to use the platforms in passive or active mode covers different exercises performed in clinical practice, such as balance (covering different components), core stability, trunk control, strengthening, limits of stability, sit to stand. In each exercise, different parameters can be set to modify the exercise difficulty depending on a patient's needs. The most relevant parameters are: the range of motion of the platform namely the degrees that the platform can reach in each direction (forward, backward, left, and right), the velocity of platform movements (when the platforms are in passive mode), the level of instability of the platform (when the platforms are in active mode with elastic or viscous dynamic resistance). All the parameters that can be changed in each exercise are reported in Table S1.

Graphic applications match the proposed exercises, promoting the patient's interaction with the device and motivating them to complete the task. The graphic applications are processed in the remote desktop.

### Training

Subjects were randomized in two groups: the control group underwent a traditional rehabilitative program with physical therapists using common rehabilitation instruments, while the experimental group performed robot-assisted rehabilitative exercises, with the constant supervision of a physical therapist.

For both groups, the treatment consisted of 15 sessions of training (three times per week, see Figure 1A), each one lasting 45 min in each session, all the activities were performed, according to phases program (Figure 1): each activity had a maximum duration of 3 min, then 30 s of resting were included after each activity. Each session was personalized according to the clinical and functional characteristics of the patient: exercise parameters were set in line with each subject's impairment and their performance during training, in order to match their specific needs and to provide a training proportional to their capabilities, but also sufficiently challenging (i.e., neither too easy nor too difficult).

Training was focused on three components of balance: steady state, proactive balance, reactive balance (13). For each of these components, focused activities were performed for both groups. In detail, steady state activities were focused on posture maintenance in sitting or standing position with static or unstable platform and seat. Proactive balance activities included: upper limbs motor tasks while maintaining balance on a static or unstable platform/seat; execution of task in asymmetric two legs or one leg load; head and trunk rotations; reaching movements and limits of stability. Reactive balance activities included postural adaptation following perturbation exercises, and upper limb tasks with an unstable seat/platform.

In order to ensure, from a functional point of view, training equivalence between the two groups, exercises were matched, i.e., the two protocols, traditional and experimental, were equally demanding in terms of duration and functional abilities addressed with training. Exercises executed on the robotic device included a graphic interface with visual and audio feedback of the subjects performance during the task (load on the platform/seat, angular displacement of the platform/seat, trunk position in sagittal and frontal plane). In addition, exercise difficulties were set depending on appropriate challenge level to avoid boredom or frustration. These aspects allowed the training to be intense, personalized and challenging. Details on how exercises were performed and their feedback in both groups are reported in Table S1.

Training was composed of three phases of five sessions (see Figure 1A) including exercises with different functional goals and level of difficulty. Phase 1 focused on steady state and proactive balance exercises. Phase 2 combined steady state, proactive and reactive balance activities. Phase 3 consisted of proactive and reactive balance activities. This treatment design was conceived to have a progression to more challenging activities over the course of training (60). A summary of the training protocol performed on the robotic device is reported in Figure 1B.

### Assessment

Participants of both groups were evaluated at T0, T1, and T2 with clinical and robot-based assessments.

#### Clinical Evaluation

An expert clinician blind to the experiment evaluated the subjects with the following tests:

- Berg Balance Scale [(52), BBS], to assess static and proactive balance through fourteen items. Each item corresponds to a specific activity with different levels of difficulty. The score for each item ranges from zero to four and the maximum score is 56;
- Trunk Impairment Scale [(61), TIS] to estimate the trunk motor impairment. The scale ranges from 0 to 23 and assesses static and dynamic postural control, as well as trunk coordination. Both the total score and the three sub-scores (static sitting balance, dynamic sitting balance, coordination) were considered in the analysis;
- Mini-BESTest [(62), MBT]. This test evaluates dynamic balance with fourteen items divided into four sub-sections: anticipatory postural adjustments; reactive postural control; sensory orientation; dynamic gait. Score for each item is between 0 and 2, for a total score of 28. Both the total score and the four sub-scores were considered in the analysis.

#### Robot-Based Evaluation

All participants were assessed with five different tests on the device.

To evaluate subjects' postural responses in different conditions, the balance performance was tested with static or dynamic tests:

- Static balance test. In this test participants stood on the platform and had to keep still and maintain their balance for 30 s, both with eyes open and with eyes closed. In this test a subject's static stability, i.e., the ability to maintain the position of the center of mass in unsupported stand when the base of support does not change, was tested. Also, with the closed eyes condition, we tested the proprioceptive and vestibular components in maintaining balance by evaluating the subjects balance performance without the support of the vision.
  For this exercise we derived sway area (95% confidence ellipse of the statokinesigram, [cm^2^]), sway path (length of the oscillation path, [cm]) and anteroposterior (AP) and mediolateral (ML) oscillation ranges of CoP expressed in [cm] (63, 64). To evaluate trunk stability we evaluated AP and ML trunk oscillations, i.e., trunk tilt in the sagittal and frontal plane [expressed in [deg], (65)] and we considered their sum to evaluate the total trunk displacement; we also computed the trunk acceleration variability, measured as the standard deviation of trunk acceleration [[deg/s^2^], (66)].
- Dynamic balance test on unstable platform. Each subject stood on the robotic platform with a low instability level. The aim of the test was to evaluate the capability of the subject to maintain balance in a dynamic situation: the platform is unstable and can move following subject's instability and oscillations, i.e., platform angular displacement is sway-referenced (67). Indeed, in this configuration, the platform angular displacement was induced by the Center of Pressure (CoP) displacement. For this exercise we analyzed the same metrics as for the static balance task, by considering the projection on a plane of the angular displacement of the platform, proportional to patient instability and oscillations: sway area (95% confidence ellipse of the statokinesigram, [cm^2^]), sway path (length of the oscillation path, [cm]) and the anteroposterior (AP) and mediolateral (ML) oscillation ranges were calculated ([cm]). As for the static balance test, to evaluate trunk stability during the test we evaluated the trunk total movement ([deg])—computed as the sum of AP and ML trunk oscillations ([deg]) and trunk acceleration variability ([deg/s^2^]).
- Reactive balance test. The subjects stood on the platform, and their capability to maintain balance in response to different perturbations was tested. The platform presented consecutively different levels of inclinations (2–4–6–8°) in one of the four cardinal directions (forward, backward, left, right, see Figure 2B). For each direction, the maximum inclination of the platform that the subjects were able to hold were determined by the therapist looking at the capability of the subjects to maintain balance with a certain degree of inclination. The exercise included a familiarization phase where the subjects standing on the platform experienced an example of how the platform could move during the actual test.
  Maximum degrees of platform inclination maintained in each direction were evaluated. Also, for each direction we measured trunk oscillations in order to evaluate the quantity of trunk movements required for subjects to maintain balance: AP and ML oscillation ranges ([deg]) were computed looking at the maximum and minimum degrees of pitch and roll angles, respectively, considering the time from the start of the perturbation up to 5 s.
  For each direction, these parameters were estimated and compared between T0 and T1 or T2 only when subjects improved or remained stable in the maximum degree reached. In order to compare trunk oscillations related to the same amount of perturbation, i.e., the same degree of platform inclination, we considered for different evaluation sessions only trials with the same base inclination, which correspond to the maximum platform inclination reached at T0.
- Proprioceptive control test (reaching task). This test was performed both in standing and in seated position. In this test subjects had to actively move the platform/seat (in standing/seated position) with feet/pelvis, respectively, while keeping the torso upright, in order to reach targets in different directions, visualized on the screen, by using their proprioceptive and vestibular control.
  This exercise assessed the ability and precision of the subject to perform selective movements with feet/pelvis to reach targets appearing randomly on the screen for 5 min.
  In particular, with this test performed in seated position, we aimed to provide a quantitative measure of the ability of the subject to stabilize the lower trunk while performing movements with the pelvis without upper trunk compensation.
  In order to assess participants' performance, we analyzed the number of reached targets, as well as changes in trunk oscillations defined as: ML, AP ranges ([deg]) and variability of the acceleration (standard deviation of trunk acceleration ([deg/s^2^]). In order to evaluate the compensatory movement of the trunk to move the platform/seat, we also computed ML and AP oscillation rate defined as the trunk oscillatory ranges normalized by the range of movement of the platform/seat in the standing/sitting trial, respectively. This ratio provided a measure of how much the patient compensated the lower limb or pelvis movement, respectively in standing or sitting positions, with the trunk. Since the request was to perform selective movements of feet or pelvis while keeping the torso upright, we expected to have good performance when this ratio was lower than 1.
  Indeed, values bigger than 1 indicated that ML or AP oscillations of the trunk were bigger than the respective indicators of the base or the seat, suggesting a compensatory strategy of the subject.
- Sit to stand. This exercise aimed at measuring the time required to reach a standing position starting from a sitting position. Subjects had to repeat the exercise three times. For this exercise, we measured: the mean duration of the three repetitions necessary to reach standing and sitting positions and the mean time to complete a sit to stand and stand to sit movement; mediolateral and anteroposterior trunk oscillations ([deg]) to evaluate control strategy during the task. These indices are computed as the maximum trunk ranges (averaged in the three repetitions) in the two planes for each movement (standing, sitting, standing and sitting together).

Patient satisfaction about rehabilitation training was measured with a visual analog scale (VAS) with a score from 0 (=no satisfaction) to 10 (=totally satisfied) at T1 evaluation session, at the end training.

### Statistical Analysis

The Mann–Whitney *U* test and a Fisher exact test were used to test the homogeneity of the groups at baseline.

Since neither clinical nor robot-based indices satisfied the normality condition (Kolmogorov-Smirnov tested), we performed non-parametric tests.

To analyze group differences, a Mann-Whitney *U* test was used to determine between-group differences. In detail, we compared the percentage of improvement of the two groups at T1 and T2, computed, respectively as (T1-T0)/T0^*^100 for T1 and (T2-T0)/T0^*^100 for T2.

To test changes in time, a Wilkoxon signed rank test was used to determine within group differences between pre- and post-training tests (T0 vs. T1 and T0 vs. T2) in each group separately. Significance level was adjusted using the Benjamini–Hochberg procedure (68) to account for multiple testing and control the false discovery rate. Consequently, the significant value was set for each parameter applying the ranking procedure. Significant results after correction are presented with a ^*^ next to the *p*-value.

All the statistical analyses have been implemented in MATLAB 2017b (MathWorks, Natick, MA, USA).

## Results

There were no significant differences between groups regarding demographic data, side of hemiparesis, stroke etiology, or outcome measures at T0 (see Table 1).

Dropout rate and reasons were similar for the two groups: a total of three subjects (1 from the experimental and 2 from the control group) dropped out of the study due to a change in their clinical/functional conditions [two subjects dropped out after the T0 evaluation, while one subject, part of the control group, did not complete the follow-up assessment (T2)]; therefore, 27 out of 30 subjects performed the whole experiment.

### Clinical Scales

Clinical indices revealed a general improvement after both the rehabilitative treatments.

**Figure 3:**
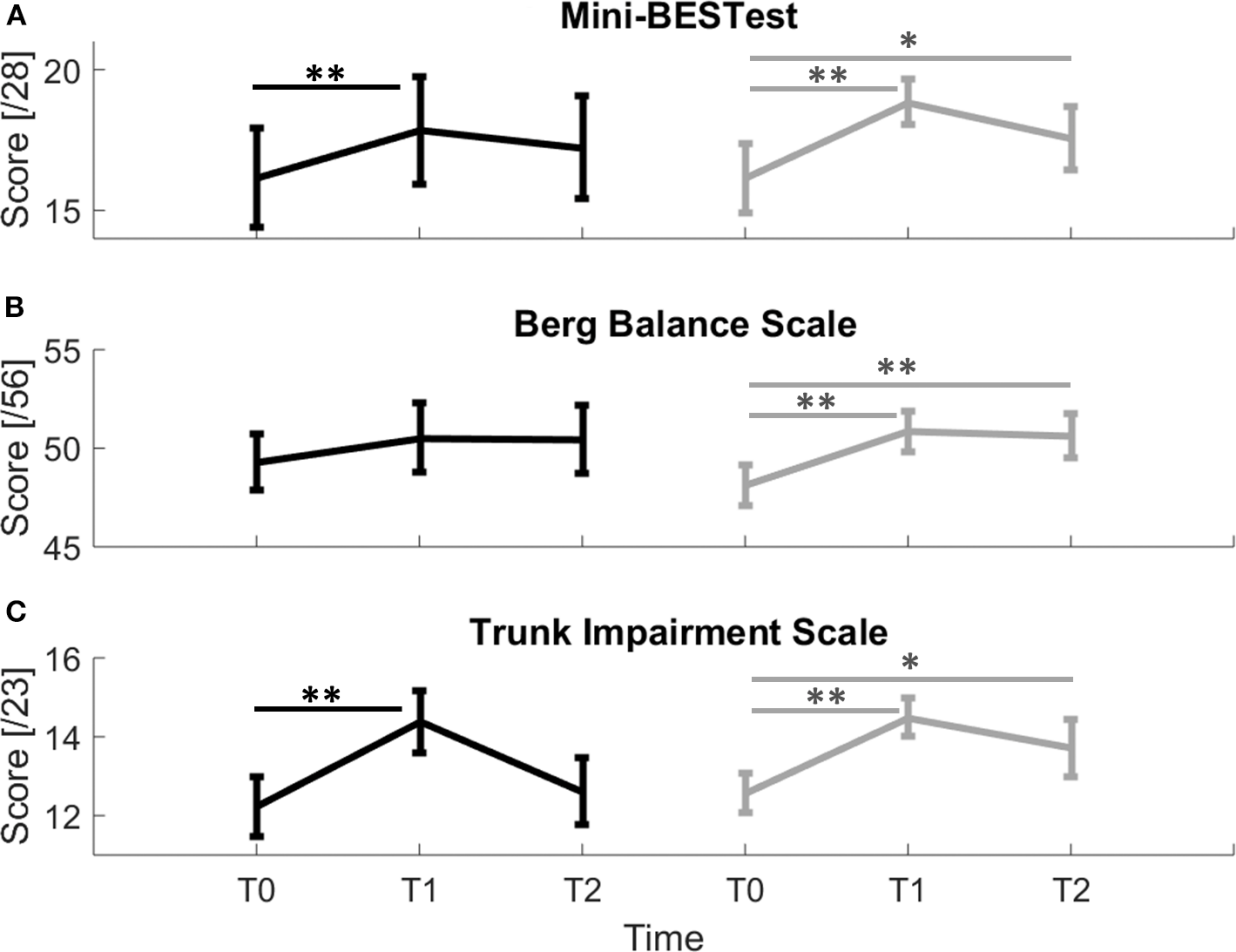
Clinical scales scores for control (black line) and experimental (gray line) groups. **(A)** Mini-BESTest; **(B)** Berg Balance Scale; **(C)** Trunk Impairment Scale. Error bars indicate Standard Error; * indicate a *p*-value between 0.01 and 0.05, while ** indicate *p* < 0.01.

Specifically, there was a significant increase after the training in both groups for the MBT (see Figure 3A and Table 2); however, only the experimental group maintained such increased at follow up (E: T0-T1: *p* < 0.001^*^ and T0-T2: *p* = 0.016^*^; C: T0-T1: *p* = 0.004^*^ and T0-T2: *p* = 0.19). In particular, the improvement in the total MBT score at T1 for the experimental group was mainly due to the combination of increase in the following sub-scores: reactive postural control (*p* = 0.047) anticipatory (*p* = 0.031), dynamic gait (*p* = 0.03) sub-scores. These parameters reached a quasi-significant level after the Benjamini & Hochberg correction. Instead, the control group significantly increased only the reactive postural control sub-score of the scale (*p* = 0.002^*^; Table 2).

**Table 2:** Mean values and statistics for BBS, TIS, and MBT and their sub-scores.

	**Groups mean values**		**Statistics**	
	**E** **Mean ± SE**	**C** **Mean ± SE**	**Time-differences** **(Wilkoxon signed rank test)**	**Group-differences** **(Mann Withney test)**
**BERG BALANCE SCALE**				
Total score [/56]	T0: 48.14 ± 0.27	T0: 49.30 ± 0.39	E: T0-T1 ***p*** = 0.003^*^	T1: *p* = 0.41
	T1: 50.85 ± 0.26	T1: 50.53 ± 0.49	E: T0-T2 ***p*** = 0.001^*^	T2: *p* = 0.6
	T2: 50.64 ± 0.29	T2: 50.46 ± 0.47	C: T0-T1 *p* = 0.07	
			C: T0-T2 *p* = 0.231	
**TRUNK IMPAIRMENT SCALE**				
Total score [/23]	T0: 12.57 ± 0.13	T0: 12.23 ± 0.21	E: T0-T1 ***p*** = 0.008^*^	T1: *p* = 0.90
	T1: 14.5 ± 0.12	T1: 14.38 ± 0.21	E: T0-T2 ***p*** = 0.045^*^	T2: *p* = 0.47
	T2: 13.71 ± 0.19	T2: 12.61 ± 0.23	C: T0-T1 ***p*** = 0.006^*^	
			C: T0-T2 *p* = 0.414	
Static sitting balance	T0: 6.35 ± 0.09	T0: 6.61 ± 0.10	E: T0-T1 *p* = 0.12	T1: *p* = 0.20
	T1: 6.85 ± 0.02	T1: 6.84 ± 0.04	E: T0-T2 *p* = 1	T2: *p* = 0.72
	T2: 6.5 ± 0.04	T2: 6.46 ± 0.10	C: T0-T1 *p* = 1	
			C: T0-T2 *p* = 0.5	
Dynamic sitting balance	T0: 4.57 ± 0.09	T0: 4 ± 0.07	E: T0-T1 ***p*** = 0.035^*^	T1: *p* = 0.31
	T1: 5.85 ± 0.11	T1: 5.84 ± 0.13	E: T0-T2 ***p*** = 0.016^*^	T2: *p* = 0.42
	T2: 5.71 ± 0.10	T2: 4.61 ± 0.11	C: T0-T1 ***p*** = 0.004^*^	
			C: T0-T2 *p* = 0.266	
Trunk coordination	T0: 1.64 ± 0.03	T0: 1.61 ± 0.06	E: T0-T1 *p* = 0.62	T1: *p* = 0.54
	T1: 1.78 ± 0.04	T1: 1.69 ± 0.09	E: T0-T2 *p* = 0.40	T2: *p* = 0.97
	T2: 1.5 ± 0.10	T2: 1.53 ± 0.10	C: T0-T1 *p* = 1	
			C: T0-T2 *p* = 0.99	
**MINI BEST TEST**				
Total score [/28]	T0: 16.14 ± 0.33	T0: 16.15 ± 0.48	E: T0-T1 ***p*** < 0.001^*^	T1: *p* = 0.32
	T1: 18.85 ± 0.21	T1: 17.84 ± 0.52	E: T0-T2 ***p*** = 0.016^*^	T2: *p* = 0.73
	T2: 17.57 ± 0.29	T2: 17.23 ± 0.50	C: T0-T1 ***p*** = 0.004^*^	
			C: T0-T2 *p* = 0.19	
Anticipatory	T0: 2.85 ± 0.07	T0: 3.15 ± 0.12	E: T0-T1 *p* = 0.031	T1: *p* = 0.10
	T1: 3.35 ± 0.05	T1: 3.15 ± 0.11	E: T0-T2 *p* = 1	T2: *p* = 0.97
	T2: 2.85 ± 0.07	T2: 3 ± 0.10	C: T0-T1 *p* = 1	
			C: T0-T2 *p* = 0.76	
Reactive post. control	T0: 2.5 ± 0.11	T0: 1.69 ± 0.11	E: T0-T1 *p* = 0.047	T1: *p* = 0.13
	T1: 3.35 ± 0.09	T1: 3.23 ± 0.13	E: T0-T2 *p* = 0.17	T2: *p* = 0.29
	T2: 3.07 ± 0.11	T2: 2.92 ± 0.15	C: T0-T1 ***p*** = 0.002^*^	
			C: T0-T2 *p* = 0.09	
Sensory orientation	T0: 4.92 ± 0.08	T0: 4.69 ± 0.13	E: T0-T1 *p* = 0.35	T1: *p* = 0.31
	T1: 5.21 ± 0.05	T1: 4.69 ± 0.14	E: T0-T2 *p* = 1	T2: *p* = 0.58
	T2: 5 ± 0.07	T2: 4.46 ± 0.15	C: T0-T1 *p* = 1	
			C: T0-T2 *p* = 0.65	
Dynamic gait	T0: 5.85 ± 0.14	T0: 6.61 ± 0.19	E: T0-T1 *p* = 0.031	T1: *p* = 0.11
	T1: 6.92 ± 0.09	T1: 6.76 ± 0.18	E: T0-T2 *p* = 0.18	T2: *p* = 0.78
	T2: 6.64 ± 0.12	T2: 6.84 ± 0.13	C: T0-T1 *p* = 0.76	
			C: T0-T2 *p* = 0.43	

E, experimental; C, control; bold values indicate significance (p < 0.05); for time comparisons

* *indicate significance considering Benjamini–Hochberg correction for multiple testing*.

Moreover, the experimental group showed a statistical improvement in the BBS scale that was maintained at follow up (Figure 3B and Table 2, E: T0-T1: *p* = 0.003^*^, T0-T2: *p* = 0.001^*^; C: T0-T1: *p* = 0.07; T0-T2: *p* = 0.231).

For the experimental group, the TIS improved after the rehabilitation and the effect was maintained at the 3 months follow-up (Figure 3C and Table 2, E: T0-T1: *p* = 0.008^*^, T0-T2: *p* = 0.045^*^). The control group significantly improved trunk control but this increased ability wasn't maintained at T2 (Figure 3C and Table 2, C T0-T1: *p* = 0.006^*^, T0-T2: *p* = 0.414).

Improvement in Trunk Impairment Scale was mainly due for both groups to an improvement in Dynamic sitting balance sub-score that only the experimental group maintained at follow up (E: T0-T1: *p* = 0.035^*^, T0-T2: *p* = 0.016^*^; C: T0-T1: *p* = 0.004^*^; T0-T2: *p* = 0.266, Table 2).

The Mann Whitney test did not reveal any group difference for all the clinical scales and their sub-scores.

Individual subjects' improvements are reported in Table S2.

### Instrumented Evaluation

#### Balance Evaluation

##### Dynamic balance on unstable platform

During this task, subjects had to maintain balance while standing on an unstable platform.

**Figure 4:**
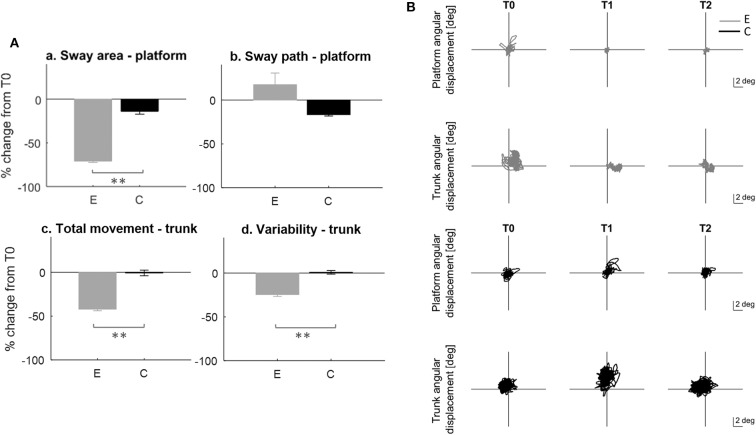
Dynamic balance test on unstable platform. **(A)** Rate of improvement T0-T1; E, experimental group; C, control group. a: sway area; b: sway path; c: trunk total angular displacement; d: standard deviation of the trunk acceleration. Error bars indicate standard error; * indicates a *p*-value between 0.01 and 0.05, while ** indicates *p* < 0.01. **(B)** Example of platform (rows 1, 3) and trunk (rows 2, 4) angular displacement raw data for two subjects, one from the experimental (gray line) and one from the control (black line) group at T0, T1, T2.

We found group differences in favour of the experimental group both in platform and trunk control parameters (Figure 4A).

Specifically, the experimental group showed at T1 improvement in sway area (between-group difference *p* < 0.001; T0-T1 E: *p* < 0.001^*^), trunk total movement (between group difference *p* < 0.001; T0-T1 E: *p* = 0.002^*^) and variability (between-group difference *p* = 0.004; T0-T1 E: *p* = 0.006^*^). Also, these improvements were maintained at T2 (T0-T2 E: sway area *p* < 0.001^*^; trunk total movement *p* = 0.002^*^; trunk variability *p* = 0.024^*^).

No differences were found between the two groups in sway pathlength; however, only the control group showed a significant decrease of this parameter that was maintained at follow up (C: T0-T1: *p* = 0.01^*^; T0-T2: *p* < 0.001^*^). All data are reported on Table 3.

**Table 3:** Parameters and statistics results for the dynamic balance test on unstable platform.

**Variables**	**Groups mean values**		**Statistics**	
	**E** **Mean ± SE**	**C** **Mean ± SE**	**Time-differences** **(Wilkoxon signed rank test)**	**Group-differences** **(Mann Withney test)**
**Balance on unstable platform**				
Sway area —platform [cm^2^]	T0: 39.55 ± 2.9	T0: 28.53 ± 2.67	E: T0-T1 ***p*** < 0.001^*^	T1: ***p*** **< 0.001**
	T1: 10.21 ± 0.73	T1: 26.08 ± 3.26	E: T0-T2 ***p*** < 0.001^*^	T2: ***p*** **= 0.002**
	T2: 12.57 ± 1.18	T2: 15.74 ± 1.17	C: T0-T1 *p* = 0.365	
			C: T0-T2 ***p*** = 0.019^*^	
Sway path [cm] —platform	T0: 139.42 ± 9.32	T0: 171.79 ± 8.08	E: T0-T1 *p* = 0.083	T1: *p* = 0.46
	T1: 98.76 ± 4.36	T1: 137.19 ± 5.20	E: T0-T2 *p* = 0.123	T2: *p* = 0.46
	T2: 97.82 ± 4.24	T2: 134.18 ± 6.55	C: T0-T1 ***p*** = 0.017^*^	
			C: T0-T2 ***p*** < 0.001^*^	
Trunk total movement [deg]	T0: 18.01 ± 0.78	T0: 14.82 ± 0.41	E: T0-T1 ***p*** = 0.002^*^	T1: ***p*** **< 0.001**
	T1: 9.85 ± 0.40	T1: 13.91 ± 0.46	E: T0-T2 ***p*** = 0.002^*^	T2: ***p*** **= 0.010**
	T2: 9.3 ± 0.25	T2: 12.19.41 ± 0.37	C: T0-T1 *p* = 0.401	
			C: T0-T2 *p* = 0.087	
Variability-trunk [deg/s^2^]	T0: 0.09 ± 0.004	T0: 0.09 ± 0.002	E: T0-T1 ***p*** = 0.006^*^	T1: ***p*** **= 0.004**
	T1: 0.06 ± 0.001	T1: 0.09 ± 0.003	E: T0-T2 ***p*** = 0.024^*^	T2: ***p*** **= 0.009**
	T2: 0.06 ± 0.001	T2: 0.09 ± 0.004	C: T0-T1 *p* = 0.96	
			C: T0-T2 *p* = 0.24	

E, experimental; C, control; bold values indicate significance (p < 0.05); for time comparisons

* *indicate significance considering Benjamini–Hochberg correction for multiple testing*.

Figure 4B report the behavior of two representative subjects, one for each group.

##### Reactive balance

During this exercise participants had to maintain balance on a progressively inclined platform. Most of the subjects were able to manage the maximum degrees of inclination already at T0 [forward: 18 (E:8; C:10); backward: 15 (E:7; C:8); affected side: 19 (E:9; C:10); not-affected side: 17 (E:10; C:7)] and maintained their performance at T1. Some subjects improved their performance at T1 with respect to T0 [forward: 5 (E:4; C:1); backward: 7 (E:5; C:2); affected side: 5 (E:3; C:2); not-affected side: 7 (E:2; C:5)]. Only a few subjects did not improve or decrease their performance (details are reported in Table S3).

**Figure 5:**
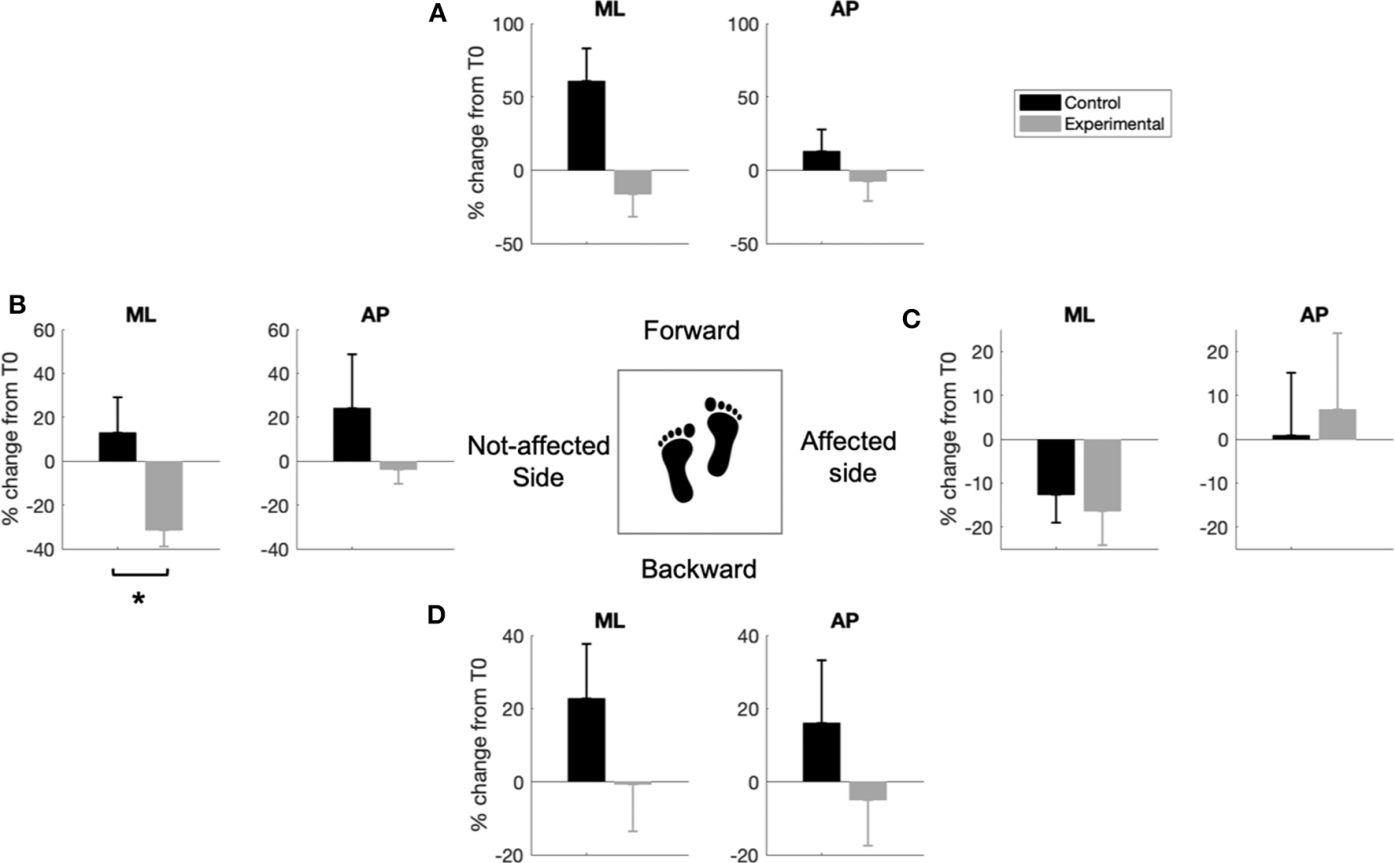
Rate of improvement T0-T1 for the reactive balance test. Each bar plot represents the percentage change of the mediolateral (ML) or anteroposterior (AP) trunk oscillatory range when the platform is inclined in the forward **(A)**, backward **(D)**, affected **(C)**, and non-affected **(B)** side of the body, for the control (black) and experimental (gray) groups. Error-bars indicate standard error, * highlight a significant difference between groups *p* < 0.05.

Looking at trunk control parameters, we found a significant group difference for the mediolateral oscillation range for perturbations provided in the not affected side direction (between groups difference *p* = 0.01, Figure 5). Specifically, the control group increased its range of oscillation, while the experimental subjects decreased it (E: T0-T1 −1.75 ± 0.14 SE [deg] *p* = 0.003^*^). This improvement was maintained at T2 (E: T0-T2 −1.9 ± 0.16 [deg] *p* = 0.007^*^). No difference between groups was found for trunk oscillations in the sagittal plane and for other directions of perturbations. To be noticed, only the experimental group significantly improved in the mediolateral range of oscillation when the perturbation was provided in the affected side (E: T0-T1 −0.80 ± 0.09 SE [deg] *p* = 0.01^*^).

##### Static balance

This exercise did not show any difference between control and experimental patients in both the eyes open and closed trials.

#### Proprioceptive Control (Reaching Task)

The proprioceptive test consisted in a reaching task where subjects were asked to reach the maximum number of targets, by mobilizing the seat or the base, in a 5-min period.

**Figure 6:**
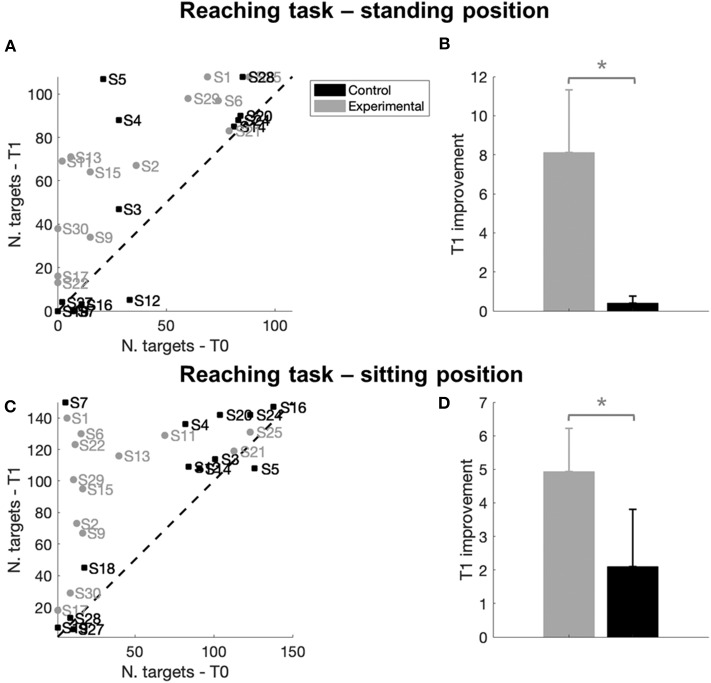
Reaching task results. **(A–C)** Number of targets reached at T0 and T1 for each subject in the standing **(A)** or sitting **(C)** trial. Different colors represent different groups: gray indicates a subject of the experimental group while black represents a subject of the control group. The dashed line represents the line of equality, where the number of targets reached before (T0) and after the treatment (T1) was identical. A data point located above the equality line (in the upper left side of the graph), indicates that a subject reached a bigger number of targets at the end of the treatment T1 than at T0, thus indicating an improvement in movement control. The opposite would hold for a point under the equality line (in the lower right side of the graph). **(B–D)** Improvement at T1 with respect to T0 in the number of reached targets in a 5-min period computed as T1-T0/T0, respectively in the standing **(B)** or sitting **(D)** trial. Error bars indicate Standard Errors; * indicate *p* < 0.05.

Figure 6A shows the number of reached targets for all subjects, with each individual number of targets reached at T1 being plotted against the same parameter at T0. All data points of the experimental group (gray dots) are distributed in the upper part of the graph, above the line of equality, indicating that after the treatment these subjects improved their performance, reaching more targets. As regards the control group, only four subjects out of 13 (30%) increased the number of reached targets; the rest of them (ten subjects, 70%) are on the line of equality or under it, indicating no improvement or a worsening in the execution of the task.

As suggested by visual inspection, we found a significant between-group difference at T1 in the performance of the two groups (*p* = 0.03, Figure 6B and Table 4), due to a significant improvement in the experimental group that reached more target at T1 (improvement T0-T1, E: *p* < 0.001^*^, Table 4) with respect to the control group. This improvement was maintained at T2 (improvement T0-T2, E: *p* = 0.010^*^, Table 4).

**Table 4:** Parameters and statistics results for the proprioceptive control test.

**Variables** **(Sensor)**	**E** **Mean ± SE**	**C** **Mean ± SE**	**Time-differences** **(Wilkoxon signed rank test)**	**Group-differences** **(Mann Withney test)**
**REACHING—STANDING POSITION**				
Number of targets	T0: 35.15 ± 2.66	T0: 36.61 ± 2.67	E: T0-T1 ***p*** < 0.001^*^	T1: ***p*** **= 0.03**
	T1: 67.61 ± 2.52	T1: 49.07 ± 3.59	E: T0-T2 ***p*** = 0.010^*^	T2: *p* = 0.63
	T2: 62.69 ± 2.80	T2: 56.53 ± 3.36	C: T0-T1 *p* = 0.278	
			C: T0-T2 ***p*** = 0.039^*^	
Variability-trunk [deg/s^2^]	T0: 0.21 ± 0.01	T0: 0.25 ± 0.01	E: T0-T1 ***p*** = 0.002^*^	T1: ***p*** **= 0.005**
	T1: 0.15 ± 0.01	T1: 0.25 ± 0.01	E: T0-T2 ***p*** = 0.003^*^	T2: *p* = 0.39
	T2: 0.16 ± 0.01	T2: 0.19 ± 0.01	C: T0-T1 *p* = 0.893	
			C: T0-T2 *p* = 0.216	
Normalized range ML—trunk	T0: 3.00 ± 0.07	T0: 3.2326 ± 0.20	E: T0-T1 ***p*** = 0.008^*^	T1: *p* =0.11
	T1: 2.31 ± 0.04	T1: 2.7576 ± 0.08	E: T0-T2 *p* = 0.296	T2: *p* = 0.94
	T2: 2.69 ± 0.06	T2: 2.67 ± 0.10025	C: T0-T1 *p* = 0.946	
			C: T0-T2 *p* = 0.305	
Normalized range AP—trunk	T0: 5.91 ± 0.12	T0: 5.63 ± 0.16	E: T0-T1 *p* = 0.104	T1: *p* = 0.27
	T1: 4.89 ± 0.14	T1: 5.68 ± 0.13	E: T0-T2 *p* = 0.808	T2: *p* = 0.48
	T2: 5.91 ± 0.16	T2: 4.73 ± 0.15	C: T0-T1 *p* = 0.893	
			C: T0-T2 *p* = 0.126	
**REACHING—SITTING POSITION**				
Number of targets	T0: 35.46 ± 3.15	T0: 69.76 ± 3.98	E: T0-T1 ***p*** < 0.001^*^	T1: ***p*** **= 0.01**
	T1: 98.76 ± 3.06	T1: 95.38 ± 4.30	E: T0-T2 ***p*** < 0.001^*^	T2: *p* = 0.16
	T2: 100.07 ± 2.82	T2: 111.84 ± 3.49	C: T0-T1 ***p*** = 0.008^*^	
			C: T0-T2 ***p*** = 0.002^*^	
Variability-trunk [deg/s^2^]	T0: 0.13 ± 0.01	T0: 0.1384 ± 0.01	E: T0-T1 *p* = 0.042	T1: ***p*** **= 0.02**
	T1: 0.09 ± 0.01	T1: 0.1413 ± 0.01	E: T0-T2 *p* = 0.058	T2: *p* = 0.68
	T2: 0.10 ± 0.01	T2: 0.11997 ± 0.01	C: T0-T1 *p* = 0.735	
			C: T0-T2 *p* = 0.111	
Normalized range ML—trunk	T0: 3.76 ± 0.09	T0: 3.27 ± 0.06	E: T0-T1 *p* = 0.173	T1: ***p*** **= 0.008**
	T1: 3.20 ± 0.06	T1: 4.21 ± 0.08	E: T0-T2 *p* = 0.119	T2: ***p*** **= 0.035**
	T2: 3.18 ± 0.09	T2: 3.66 ± 0.07	C: T0-T1 ***p*** = 0.001^*^	
			C: T0-T2 *p* = 0.168	
Normalized range AP—trunk	T0: 8.36 ± 0.20	T0: 7.80 ± 0.18	E: T0-T1 ***p*** = 0.003^*^	T1: ***p*** **= 0.039**
	T1: 5.48 ± 0.10	T1: 6.91 ± 0.15	E: T0-T2 ***p*** = 0.003^*^	T2: ***p*** **= 0.021**
	T2: 5.54 ± 0.10	T2: 7.69 ± 0.17	C: T0-T1 *p* = 0.414	
			C: T0-T2 *p* = 0.946	

E, experimental; C, control; bold values indicate significance (p < 0.05); for time comparisons

* *indicate significance considering Benjamini–Hochberg correction for multiple testing*.

**Figure 7:**
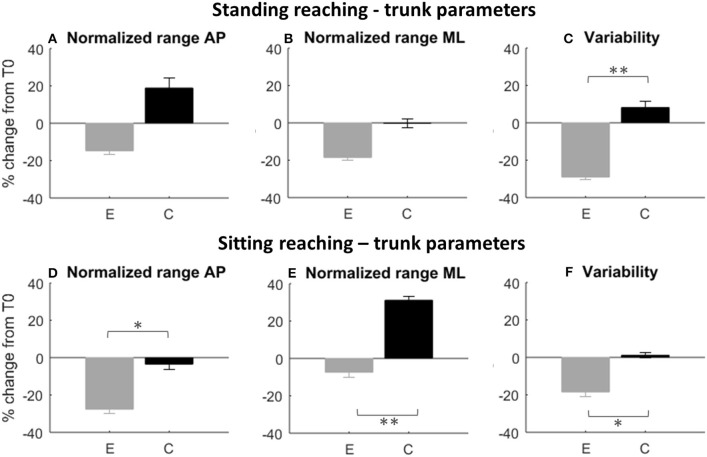
Rate of improvement T0-T1 for the proprioceptive control test. Trunk oscillation changes during the reaching task in standing (first row) and sitting (second row) position are represented. E, experimental group; C, control group. **(A–D)** Anteroposterior (AP) oscillatory range of the trunk; **(B–E)**: mediolateral (ML) oscillatory range of the trunk; **(C–F)**: standard deviation of the trunk acceleration. Error bars indicate standard error; * indicate a *p*-value between 0.01 and 0.05, while ** indicate *p* < 0.01.

Looking at trunk control during the task, we found a significant difference in trunk variability between the two groups (*p* = 0.005, Figure 7C), with a significant decrease of trunk movements during the task only for the experimental group at T1 (T0-T1, E: *p* = 0.002^*^, Table 4) and T2 (T0-T2, E: *p* = 0.003^*^, Table 4). There were no differences between groups in the other parameters (see Figures 7A,B); however, only the experimental group showed a T1 decrease of the trunk oscillation range in the frontal plane (T0-T1, E: *p* = 0.008^*^, Table 4).

As for the sitting test, almost all the subjects of the two groups improved the number of targets reached. In Figure 6C, 26 data points out of 27 are located above the line of equality, suggesting a general improvement in the execution of the task. More in detail, the gray data points that represent experimental group were located in the upper part of the graph, highlighting a greater improvement. Indeed, we found a between group difference in task performance at T1 (*p* = 0.01, Figure 6D and Table 4) due to a greater improvement in the experimental group compared to the control group (improvement T0-T1, E: *p* < 0.001^*^; C: *p* = 0.008^*^, Table 4); both groups maintained the improvement at T2 (improvement T0-T2, E: *p* < 0.001^*^; C: *p* = 0.002^*^, Table 4).

Experimental group also improved in normalized trunk range of oscillation in the anteroposterior direction (between group difference *p* = 0.039, Figure 7D; improvement T0-T1, E: *p* = 0.003^*^). The decreased trunk oscillation in sagittal direction during the sitting reaching task was maintained at T2 (between group difference *p* = 0.021, improvement T0-T2, E: *p* = 0.003^*^).

Moreover, the control group increased significantly the normalized trunk oscillation range in the mediolateral direction (between group difference *p* = 0.008^*^, Figure 7F, T0-T1 C: *p* = 0.001^*^). A significant between group difference was found in trunk variability at T1 (*p* = 0.02, Figure 7F), but without significant changes in time for both groups.

All data are reported in Table 4.

#### Sit to Stand Evaluation

At T0, nine subjects (five subjects from the experimental group and four from the control group) were not able to complete the task. However, at T1 three of them, part of the experimental group, succeeded in completing the task.

Performance in the task was compared between the two groups considering only participants who performed the sit to stand both at T0 and T1.

We did not find any group difference, even though the experimental group showed a statistically significant improvement in the mean sit to stand time (improvement T0-T1, E: −2.99 ± 0.30 SE [s], *p* = 0.031^*^; C: −1.35 ± 0.16 SE [s], *p* = 0.039) that was preserved after 3 months (T0-T2, E: −1.97 ± 0.22 SE [s], *p* = 0.031^*^, C: −0.76 ± 0.19 SE [s], *p* = 0.130).

### Patients Satisfaction

At the end of the treatment both groups showed a good satisfaction about the rehabilitative training performed. Satisfaction score for the experimental group was greater, despite the group difference not reaching statistical significance (mean VAS at T1, E: 9.57 ± 0.20 SE; C: 8.92 ± 0.30 SE).

## Discussion

Stroke survivors typically present balance and core stability impairments.

Rehabilitative interventions usually include exercises focused on trunk control and balance, functional activities that are directly correlated with gait recovery and rehabilitation.

Even if several studies report improvements after balance training in stroke survivors (69), there is still poor evidence supporting the effectiveness of a specific training (12). Repetitive task training, virtual reality and training with unstable support surface have been reported to positively affect balance in stroke patients.

In this context, robotic devices are novel and powerful instruments for stroke rehabilitation, due to their adaptability to a patient's impairment, repeatability and training intensity. Moreover, robotic devices can provide biofeedback on subject's performance.

In this study, we evaluated the effect of core stability and balance rehabilitative training on quantitatively assessed balance and trunk stability, performed with a novel robotic device compared to a traditional treatment. Both treatments (robotic and control) were focused on steady state, proactive and reactive components of balance, working on balance and trunk control.

Our results show that both rehabilitative programs were effective in improving balance and trunk stability for chronic stroke patients, as suggested by the improvements of functional clinical scales at the end of the treatment. However, instrumented evaluation highlighted a greater improvement in the experimental group in the dynamic balance (balance on unstable and perturbating platform) and proprioceptive tasks, with an improved trunk control and reduced compensatory strategies.

Moreover, the experimental group showed a better retention of the improvements at 3 months follow up.

### Balance and Trunk Stability Training Is Effective in Chronic Stroke Patients

The performed training led to improvements for both groups on trunk control and balance. Specifically, experimental and control groups showed greater MBT scores after treatment.

The mean improvement in the MBT for the experimental group was 2.71 (23.65% from baseline), close to the Minimal Detectable Change (MDC) for stroke subjects [three points (70)], while it was lower for the control group (+1.69, 10.40% from baseline). Moreover, only the experimental group maintained this improvement at 3 months follow up.

Looking at individual improvements, a total of nine subjects showed an improvement >2.8 (10% of the total score); five of them were from the experimental group and showed a medium-low baseline score (lower than 11) except for one subject that had a baseline score of 17. Subjects with a score higher than 17 presented a null or very small (=1) T1 improvement.

Interestingly, while for the experimental group the MBT improvement was mainly due to the combination of increase in the reactive postural control, dynamic gait and anticipatory sub-scores, for the control group this improvement was due to a significant improvement in the reactive postural control sub-score. These results highlight how the training was in general more effective in improving dynamic balance components rather than static ones. This was further supported by the lack of static balance improvements in both groups. Intriguingly, only the experimental group showed improvements in the anticipatory and dynamic gait sub-scores of MBT.

As for the BBS scale, we did not find significant differences in the behavior of the two groups; however, only the experimental group showed a significant improvement, maintained at follow up, in this scale. In detail, the BBS score increase for the experimental group was about 2.71 points (5.89% from the baseline evaluation, vs. the 2.37% of the control group). Even if such improvement did not reach the MDC threshold [5 points, 10% (71)], it was in line with other studies investigating the effects of treatment on balance (evaluated with BBS) in chronic stroke survivors (72). Indeed, BBS tends to have greater responsiveness to changes earlier after stroke (73), showing significant ceiling effects for patients with mild stroke impairments with the possibility to miss significant gains in balance that are critically important for quality of life and community reintegration (74). Also, it is important to consider that the mean value of the score at baseline was already high for both groups (48.14 ± 0.27 SE for the experimental and 49.30 ± 0.39 SE for the control group); nevertheless both groups exceeded the threshold of 49 that is indicated as fall risk threshold (75) for stroke survivors: only 12 subjects (eight from the experimental group) out of 27 had a score lower than 49 at baseline, 8 (seven from the experimental group) improved the score and 4 (all from the experimental group) exceeded the threshold of 49 after the training.

Looking at individual values, only five subjects had an improvement >5.6 (10% of the total score); three of them were part of the experimental group and presented a score lower than 48 at baseline. Indeed, for the experimental group subjects with baseline scores superior or equal to 48 (*n* = 7) had a null or small improvement.

In our study both groups improved in the TIS score. The mean improvement for the total score was for both groups close to two points, the 10% of the maximum score. In total 11 subjects had an improvement >2.3 (10% of the total score), six were from the experimental group and showed a baseline score lower than 14. TIS scale increases were mainly due to better dynamic sitting balance sub-score; this was in accordance with a previous study (76) that found an improved dynamic sitting balance after a training focused on core stability. A group of studies already reported that unstable conditions are effective in stroke rehabilitation, especially in sitting balance (77). As such, unstable surface has been reported to increase trunk flexibility, muscular activity, strength, dynamic balance, endurance and proprioception (78, 79). Both treatments were effective in improving trunk control but only the experimental group maintained such improvement at follow up, suggesting that the robotic training led to a greater retention.

The enhanced balance and trunk control led to improvements in postural transitions, as measured by the sit to stand test performed with the robotic device: all subjects decreased the time to stand, a parameter that strongly correlates with gait performance (80). It is worth noting that, after training, some subjects of the experimental group that were not able to perform the task at the beginning of the training, succeeded in performing the task.

Even if several studies show improvement in chronic stroke survivors after balance or core stability training (28, 33), our study adds some knowledge in the field. We already know that robot-based systems can provide interactive and personalized programs maximizing the impact of rehabilitation through highly repetitive and quantifiable exercises (36, 81, 82), but to the best of our knowledge the device used in this study is the first robotic device focused on balance, core stability and trunk control rehabilitation. Our results show how this system is completely feasible in rehabilitation of chronic stroke survivors; moreover, the robot assisted rehabilitation program was at least comparable with the one performed by the control group, addressing all the components of steady state, proactive and reactive balance.

Interestingly, only the experimental group maintained the training-related improvement achieved at 3 months follow-up. A possible explanation is that the robotic aided therapy, provided more intense, challenging, personalized and, as a consequence, more engaging training, inducing to a greater retention.

### Robotic Treatment Increases Trunk Stability While Decreasing Compensatory Strategies

One of our initial hypotheses was that, with the robotic training, subjects could improve trunk control. During the robot-assisted activities, a continuous visual and audio feedback about trunk control and compensation during balance or core stability tasks was given.

After training, the experimental group showed greater improvements in the proprioceptive-reaching test in the seated and standing position. In particular, subjects belonging to the experimental condition reached a higher number of targets controlling platform or seat with pelvis or lower limbs; moreover, they showed a better control of the upper body, as they compensated less with the trunk. This means that subjects learned to perform the task with a better and more efficient control strategy.

In seated position, subjects could reach more targets showing an increased pelvis range of movement. This was in line with Haruyama et al. (28) who found an increased pelvis range of motion after a training focused on core stability with selective pelvic exercises. Moreover, subjects reduced the compensatory movements of the trunk.

Stroke survivors often compensate postural and motor deficits exploiting the redundancy of the motor system, i.e., developing “compensatory strategies” that may involve trunk movements and posture (83, 84); these new motor patterns often represent in chronic stroke survivors a well-established behavior, strengthened with time (83). True recovery is defined as the restoration of functions and abilities as they were before injury. This definition is in contrast with the concept of compensation occurring when alternative brain areas are activated in the execution of a task, producing movements performed in a new way and with different parts of the body compared to before injury (84). In terms of performance and functional level, true recovery means to (i) restore the same physical abilities that were present before the acute event (i.e., correct joint coordination and muscle activations) and (ii) complete a task in the same functional manner as an unimpaired person.

Even though trunk stability can be improved with different type of exercises, it is still not clear whether patients uses compensatory strategies or whether the motor function is properly recovered (84). A recent review on upper-limb robotic systems reported that such devices can enhance true recovery rather than compensation (81).

Due to poor core stability, pelvis movements were associated with trunk compensatory strategies (24). After the robotic treatment, we found that the experimental group was able to decrease these strategies by disassociating pelvis movements and upper trunk stabilization, improving joint and body regions coordination during the task.

### Robotic Treatment Improves Dynamic Balance

After training, the group trained with the robotic device showed a better balance control in the dynamic condition while standing on the unstable platform; subjects were able to better control the platform's oscillation while reducing trunk movements with respect to the control group. Previous studies showed how core stabilization exercises can improve dynamic balance in stroke subjects (28, 33, 35). Core training possibly improved the balance of the lumbo-pelvic-hip complex, correcting postural alignment, and increasing balance of the whole body.

Moreover, in the reactive balance test the experimental group presented an enhanced control strategy after treatment. When the perturbation was provided on the unaffected side, subjects of the experimental group were able to react to perturbation with less movements of the trunk. Indeed, our data showed a significant group difference in the mediolateral oscillation range for perturbations provided in the direction of the unaffected side. Also, the experimental group decreased mediolateral oscillations of the trunk when the perturbation was provided in the direction of the affected side. These results show that by the exposure to robotic perturbations, participants in the experimental group were able to find and learn a more efficient strategy to control their posture after a sudden perturbation compared to the control group. Our results are in line with an RCT in chronic stroke patients (72) showing significant improvements in reactive subscale of the MBT in the group that received a perturbation-based balance training.

Results of the clinical scales did not detect group differences, despite robot-based tests investigating dynamic balance suggest a better improvement of the experimental group, compared to the controls.

A possible explanation of this divergence could be that clinical scales are not always able to detect compensatory strategies (85). Both groups improved in the reactive balance score of the MBT, and this was in line with fact that they showed similar capability of managing different degrees of platform inclination in the reactive balance test on the robotic device. However, when we looked at the reaction strategy, i.e., the trunk oscillations following perturbation, we found a different behavior comparing the two groups. Moreover, we have to notice that, even if we did not find significant between-group differences in these tests, only the experimental group reached significant improvements after treatment in BBS, and MBT dynamic gait and anticipatory scores.

### Conclusions

We believe this is the first study assessing the role of a robot-based rehabilitative device in improving balance and trunk stability of stroke patients.

Our findings support the introduction of balance and core stability training to improve trunk and mobility dysfunctions in chronic stroke patients.

Balance and trunk stability training based on a robotic graded protocol is beneficial to trunk control, as well as to dynamic reactive balance measures leading to a better retention of the improvements at 3 months follow up. To this extent, we can expect that a balance and trunk stability training in subacute stroke patients, who are in the process of neurological recovery and present less consolidate compensatory strategies, could bring greater functional recovery.

### Limitation of the Study

This study reports a preliminary experience of core stability and balance training performed with hunova in chronic stroke survivors. In order to confirm these preliminary results, the sample size should be increased. Since it was not feasible to perform a large study with a heterogeneous population, we chose a narrow inclusion criteria for a homogenous group of only chronic high functioning stroke survivors.

Moreover, the improvements we reported were assessed through instrumented evaluations and clinical scales (i.e., BBS and TIS and MBT). The introduction of outcomes, such as walking speed or distance would have provided additional information about the impact of the observed improvements on relevant activities of daily life.

Thus, for a generalization of the obtained results, the study should be repeated in a larger sample of patients using additional more functional and participation outcomes.

Lastly, in this study we compared one group which performed activities with the physical therapist with a group performing the same activities with a robotic device. Specifically, the activities performed by the two groups were matched from a functional point of view. This could be a limitation as it does not completely reflect the clinical practice, reducing the potential of the robot-based training.

## Data Availability Statement

The raw data supporting the conclusions of this article will be made available by the authors, without undue reservation, to any qualified researcher.

## Ethics Statement

The studies involving human participants were reviewed and approved by Liguria Regional Ethics Committee (CER Liguria). The patients/participants provided their written informed consent to participate in this study.

## Author Contributions

GC was the principal investigator. AD, VS, LB, and HV designed the project. CL and GC recruited the subjects. LB, HV, IP, CCap, and CCas performed the robotic training. AD, SR, and VS analyzed the data and wrote the paper. LD, JS, and CS supervised the technological aspects of the project. All authors contributed to the manuscript revision, read, and approved the submitted version.

## Conflict of Interest

AD, VS, JS, and CS are employees of Movendo Technology (Genoa, Italy). The remaining authors declare that the research was conducted in the absence of any commercial or financial relationships that could be construed as a potential conflict of interest.

## References

[1] Piirtola M Era P. Force platform measurements as predictors of falls among older people–a review. Gerontology. (2006) 52:1–16. 10.1159/000089820 16439819

[2] Feigin VL Lawes CMM Bennett DA Barker-Collo SL Parag V. Worldwide stroke incidence and early case fatality reported in 56 population-based studies: a systematic review. Lancet Neurol. (2009) 8:355–69. 10.1016/S1474-4422(09)70025-0 19233729

[3] Verheyden G Vereeck L Truijen S Troch M Herregodts I Lafosse C. Trunk performance after stroke and the relationship with balance, gait and functional ability. Clin. Rehabil. (2006) 20:451–8. 10.1191/0269215505cr955oa 16774097

[4] Rasmussen-Barr E Nilsson-Wikmar L Arvidsson I. Stabilizing training compared with manual treatment in sub-acute and chronic low-back pain. Man Ther. (2003) 8:233–41. 10.1016/S1356-689X(03)00053-5 14559046

[5] Bobath B Adult Hemiplegia : Evaluation and Treatment. 3rd ed. Oxford: Heinemann Medical Books (1990).

[6] Batchelor FA Mackintosh SF Said CM Hill KD. Falls after Stroke. Int Stroke J. (2012) 7:482–90. 10.1111/j.1747-4949.2012.00796.x 22494388

[7] Eng JJ Pang MY Ashe MC. Balance, falls, and bone health: role of exercise in reducing fracture risk after stroke. J Rehabil Res Dev. (2008) 45:297–14. 10.1682/JRRD.2007.01.0014 18566947

[8] Ada L Dean CM Mackey FH Increasing the amount of physical activity undertaken after stroke. Phys Ther Rev. (2006) 11:91–100. 10.1179/108331906X98994

[9] Dean CM Richards CL Malouin F. Task-related circuit training improves performance of locomotor tasks in chronic stroke: a randomized, controlled pilot trial. Arch Phys Med Rehabil. (2000) 81:409–17. 10.1053/mr.2000.3839 10768528

[10] Teixeira-Salmela LF Olney SJ Nadeau S Brouwer B. Muscle strengthening and physical conditioning to reduce impairment and disability in chronic stroke survivors. Arch Phys Med Rehabil. (1999) 80:1211–8. 10.1016/S0003-9993(99)90018-7 10527076

[11] Pang MY Eng JJ Dawson AS McKay HA Harris JE. A community-based fitness and mobility exercise program for older adults with chronic stroke: a randomized, controlled trial. J Am Geriatr Soc. (2005) 53:1667–74. 10.1111/j.1532-5415.2005.53521.x 16181164 PMC3226792

[12] Arienti C Lazzarini SG Pollock A Negrini S. Rehabilitation interventions for improving balance following stroke: an overview of systematic reviews. PLoS ONE. (2019) 14:e0219781. 10.1371/journal.pone.0219781 31323068 PMC6641159

[13] Shumway-Cook A Woollacott M Motor Control : Translating Research Into Clinical Practice. 3rd ed Philadelphia, PA: Lippincott Williams and Wilkins (2007).

[14] Gschwind YJ Kressig RW Lacroix A Muehlbauer T Pfenninger B Granacher U A best practice fall prevention exercise program to improve balance, strength/power, and psychosocial health in older adults: study protocol for a randomized controlled trial. BMC Geriatr. (2013) 13:105 10.1186/1471-2318-13-105 24106864 PMC3852637

[15] Muehlbauer T Besemer C Wehrle A Gollhofer A Granacher U. Relationship between strength, power and balance performance in seniors. Gerontology. (2012) 58:504–12. 10.1159/000341614 22922168

[16] Neckel ND Blonien N Nichols D Hidler J. Abnormal joint torque patterns exhibited by chronic stroke subjects while walking with a prescribed physiological gait pattern. J Neuroeng Rehabil. (2008) 5:19. 10.1186/1743-0003-5-19 18761735 PMC2553074

[17] Hsieh C-L Sheu C-F Hsueh I-P Wang C-H. Trunk control as an early predictor of comprehensive activities of daily living function in stroke patients. Stroke. (2002) 33:2626–30. 10.1161/01.STR.0000033930.05931.93 12411652

[18] Dickstein R Shefi S Marcovitz E Villa Y. Electromyographic activity of voluntarily activated trunk flexor and extensor muscles in post-stroke hemiparetic subjects. Clin Neurophysiol. (2004) 115:790–6. 10.1016/j.clinph.2003.11.018 15003758

[19] Geurts AC de Haart M van Nes IJ Duysens J. A review of standing balance recovery from stroke. Gait Posture. (2005) 22:267–81. 10.1016/j.gaitpost.2004.10.002 16214666

[20] Tanaka S Hachisuka K Ogata H. Muscle strength of trunk flexion-extension in post-stroke hemiplegic patients1. Am J Phys Med Rehabil. (1998) 77:288–90. 10.1097/00002060-199807000-00005 9715916

[21] Karatas M Çetin N Bayramoglu M Dilek A. Trunk muscle strength in relation to balance and functional disability in unihemispheric stroke patients. Am J Phys Med Rehabil. (2004) 83:81–7. 10.1097/01.PHM.0000107486.99756.C7 14758293

[22] Geiger RA Allen JB O'Keefe J Hicks RR. Balance and mobility following stroke: effects of physical therapy interventions with and without biofeedback/forceplate training. Phys Ther. (2001) 81:995–1005. 10.1093/ptj/81.4.995 11276182

[23] Verheyden G Vereeck L Truijen S Troch M Lafosse C Saeys W. Additional exercises improve trunk performance after stroke: a pilot randomized controlled trial. Neurorehabil Neural Repair. (2008) 23:281–6. 10.1177/1545968308321776 18955513

[24] Messier S Bourbonnais D Desrosiers J Roy Y. Dynamic analysis of trunk flexion after stroke1. Arch Phys Med Rehabil. (2004) 85:1619–24. 10.1016/j.apmr.2003.12.043 15468021

[25] Ryerson S Byl NN Brown DA Wong RA Hidler JM. Altered trunk position sense and its relation to balance functions in people post-stroke. J Neurol Phys Ther. (2008) 32:14–20. 10.1097/NPT.0b013e3181660f0c 18463551

[26] Mergner T Maurer C Peterka RJ. A multisensory posture control model of human upright stance. Prog Brain Res. (2003) 142:189–201. 10.1016/S0079-6123(03)42014-1 12693262

[27] Faries MD Greenwood M Core training: stabilizing the confusion. Strength Cond J. (2007) 29:10–25. 10.1519/1533-4295200729[10:CTSTC]2.0.CO2

[28] Haruyama K Kawakami M Otsuka T. Effect of core stability training on trunk function, standing balance, and mobility in stroke patients. Neurorehabil Neural Repair. (2017) 31:240–9. 10.1177/1545968316675431 27821673

[29] Key J ‘The core’ Understanding it retraining its dysfunction. J Bodyw Mov Ther. (2013) 17:541–59. 10.1016/j.jbmt.2013.03.012 24139017

[30] Hibbs AE Thompson KG French D Wrigley A Spears I. Optimizing performance by improving core stability and core strength. Sport Med. (2008) 38:995–1008. 10.2165/00007256-200838120-00004 19026017

[31] Cabanas-Valdés R Bagur-Calafat C Girabent-Farrés M Caballero-Gómez FM Hernández-Valiño M Urrútia Cuchí G. The effect of additional core stability exercises on improving dynamic sitting balance and trunk control for subacute stroke patients: a randomized controlled trial. Clin. Rehabil. (2015) 30:1024–33. 10.1177/0269215515609414 26451007

[32] Yu S-H Park S-D. The effects of core stability strength exercise on muscle activity and trunk impairment scale in stroke patients. J Exerc Rehabil. (2013) 9:362–7. 10.12965/jer.130042 24278885 PMC3836527

[33] Chung E-J Kim J-H Lee B-H. The effects of core stabilization exercise on dynamic balance and gait function in stroke patients. J Phys Ther Sci. (2013) 25:803–6. 10.1589/jpts.25.803 24259857 PMC3820398

[34] Verheyden G Nieuwboer A Van de Winckel A De Weerdt W. Clinical tools to measure trunk performance after stroke: a systematic review of the literature. Clin Rehabil. (2007) 21:387–94. 10.1177/0269215507074055 17613559

[35] Ting-Ting L Meng-Jie L Ya-Qian L Li-Na M Chang-De J Effects of core stability exercise on rehabilitation in stroke patients with hemiplegia: a meta-analysis. TMR Nondrug Therapy. (2018) 1:41–52. 10.12032/TMRND201801007

[36] Poli P Morone G Rosati G Masiero S. Robotic technologies and rehabilitation: new tools for stroke patients' therapy. Biomed Res Int. (2013) 2013:153872. 10.1155/2013/153872 24350244 PMC3852950

[37] Carey JR Bhatt E Nagpal A. Neuroplasticity promoted by task complexity. Exerc Sport Sci Rev. (2005) 33:24–31. 15640717

[38] Badke MB Duncan PW. Patterns of rapid motor responses during postural adjustments when standing in healthy subjects and hemiplegic patients. Phys Ther. (1983) 63:13–20. 10.1093/ptj/63.1.13 6849002

[39] Dettmann MA Linder MT Sepic SB. Relationships among walking performance, postural stability, and functional assessments of the hemiplegic patient. Am. J. Phys. Med. (1987) 66:77–90. 3578493

[40] Goldie PA Matyas TA Evans OM Galea M Bach TM. Maximum voluntary weight-bearing by the affected and unaffected legs in standing following stroke. Clin Biomech. (1996) 11:333–42. 10.1016/0268-0033(96)00014-9 11415642

[41] Horak FB Esselman P Anderson ME Lynch MK. The effects of movement velocity, mass displaced, and task certainty on associated postural adjustments made by normal and hemiplegic individuals. J Neurol Neurosurg Psychiatry. (1984) 47:1020–8. 10.1136/jnnp.47.9.1020 6481370 PMC1028008

[42] Shumway-Cook A Anson D Haller S. Postural sway biofeedback: its effect on reestablishing stance stability in hemiplegic patients. Arch Phys Med Rehabil. (1988) 69:395–400. 3377664

[43] Tessem S Hagstrom N Fallang B. Weight distribution in standing and sitting positions, and weight transfer during reaching tasks, in seated stroke subjects and healthy subjects. Physiother Res Int. (2007) 12:82–94. 10.1002/pri.362 17536646

[44] van Nes IJ Nienhuis B Latour H Geurts AC. Posturographic assessment of sitting balance recovery in the subacute phase of stroke. Gait Posture. (2008) 28:507–12. 10.1016/j.gaitpost.2008.03.004 18424149

[45] Wade DT Hewer RL. Functional abilities after stroke: measurement, natural history and prognosis. J Neurol Neurosurg Psychiatry. (1987) 50:177–82. 10.1136/jnnp.50.2.177 3572432 PMC1031489

[46] Goldie PA Bach TM Evans OM. Force platform measures for evaluating postural control: reliability and validity. Arch Phys MedRehabil. (1989) 70:510–7. 2742465

[47] Nichols DS Miller L Colby LA Pease WS. Sitting balance: its relation to function in individuals with hemiparesis. Arch Phys Med Rehabil. (1996) 77:865–9. 10.1016/S0003-9993(96)90271-3 8822675

[48] Hodges PW Richardson CA. Relationship between limb movement speed and associated contraction of the trunk muscles. Ergonomics. (1997) 40:1220–30. 10.1080/001401397187469 9375536

[49] Kaminski TR Bock C Gentile AM. The coordination between trunk and arm motion during pointing movements. Exp Brain Res. (1995) 106:457–66. 10.1007/BF00231068 8983989

[50] Bouisset S Zattara M. Biomechanical study of the programming of anticipatory postural adjustments associated with voluntary movement. J Biomech. (1987) 20:735–42. 10.1016/0021-9290(87)90052-2 3654672

[51] Dickstein R Shefi S Marcovitz E Villa Y. Anticipatory postural adjustment in selected trunk muscles in poststroke hemiparetic patients1. Arch Phys Med Rehabil. (2004) 85:261–7. 10.1016/j.apmr.2003.05.011 14966711

[52] Berg K Wood-Dauphine S Williams JI Gayton D Measuring balance in the elderly: preliminary development of an instrument. Physiother Canada. (1989) 41:304–11. 10.3138/ptc.41.6.304

[53] Folstein MF Folstein SE McHugh PR ‘Mini-mental state’: a practical method for grading the cognitive state of patients for the clinician. J. Psychiatr. Res. (1975) 12:189–98. 10.1016/0022-3956(75)90026-6 1202204

[54] Lopez MN Charter RA Mostafavi B Nibut LP Smith WE. Psychometric properties of the folstein mini-mental state examination. Assessment. (2005) 12:137–44. 10.1177/1073191105275412 15914716

[55] Toglia J Fitzgerald KA O'Dell MW Mastrogiovanni AR Lin CD. The mini-mental state examination and montreal cognitive assessment in persons with mild subacute stroke: relationship to functional outcome. Arch Phys Med Rehabil. (2011) 92:792–8. 10.1016/j.apmr.2010.12.034 21530727

[56] De Renzi E Faglioni P. Normative data and screening power of a shortened version of the token test. Cortex. (1978) 14:41–9. 10.1016/S0010-9452(78)80006-9 16295108

[57] Ambrosoni E Della Sala S Motto C Oddo S Spinnler H. Gesture imitation with lower limbs following left hemisphere stroke. Arch Clin Neuropsychol. (2006) 21:349–58. 10.1016/j.acn.2006.05.001 16777371

[58] Saglia JA Tsagarakis NG Dai JS Caldwell DG Control strategies for patient-assisted training using the ankle rehabilitation robot (ARBOT). IEEE/ASME Trans Mechatronics. (2013) 18:1799–808. 10.1109/TMECH.2012.2214228

[59] Saglia J De Lusa A Squeri V Ciaccia L Sanfilippo C Ungaro S. Design and development of a novel core, balance and lower limb rehabilitation robot: Hunova®. IEEE Int Conf Rehabil Robot. (2019) 2019:417–22. 10.1109/ICORR.2019.8779531 31374665

[60] Winstein CJ Stein J Arena R Bates B Cherney LR Cramer SC. Guidelines for adult stroke rehabilitation and recovery. Stroke. (2016) 47:e98–169. 10.1161/STR.0000000000000098 27145936

[61] Verheyden G Nieuwboer A Mertin J Preger R Kiekens C De Weerdt W. The trunk impairment scale: a new tool to measure motor impairment of the trunk after stroke. Clin Rehabil. (2004) 18:326–34. 10.1191/0269215504cr733oa 15137564

[62] Franchignoni F Horak F Godi M Nardone A Giordano A. Using psychometric techniques to improve the balance evaluation systems test: the mini-BESTest. J Rehabil Med. (2010) 42:323–31. 10.2340/16501977-0537 20461334 PMC3228839

[63] Paillard T Noé F. Techniques and methods for testing the postural function in healthy and pathological subjects. Biomed Res Int. (2015) 2015:891390. 10.1155/2015/891390 26640800 PMC4659957

[64] Nguyen US Kiel DP Li W Galica AM Kang HG Casey VA. Correlations of clinical and laboratory measures of balance in older men and women. Arthritis Care Res (Hoboken). (2012) 64:1895–902. 10.1002/acr.21783 22745045 PMC3467339

[65] Ghahramani M Stirling D Naghdy F Naghdy G Potter J. Body postural sway analysis in older people with different fall histories. Med Biol Eng Comput. (2019) 57:533–42. 10.1007/s11517-018-1901-5 30259474

[66] Cella A De Luca A Squeri V Parodi S Puntoni M Vallone F. Robotic balance assessment in community-dwelling older people with different grades of impairment of physical performance. Aging Clin Exp Res. (2019) 32:491–503. 10.1007/s40520-019-01395-0 31691151

[67] Nashner LM Black FO Wall C III. Adaptation to altered support and visual conditions during stance: patients with vestibular deficits. J Neurosci. (1982) 2:536–44. 10.1523/JNEUROSCI.02-05-00536.1982 6978930 PMC6564270

[68] Benjamini Y Hochberg Y Controlling the false discovery rate: a practical and powerful approach to multiple testing. J R Stat Soc Ser B. (1995) 57:289–300. 10.1111/j.2517-6161.1995.tb02031.x

[69] Hammer A Nilsagård Y Wallquist M Balance training in stroke patients–a systematic review of randomized, controlled trials. Adv Physiother. (2008) 10:163–72. 10.1080/14038190701757656

[70] Tsang CS Liao L-R Chung RC Pang MY. Psychometric properties of the mini-balance evaluation systems test (Mini-BESTest) in community-dwelling individuals with chronic stroke. Phys Ther. (2013) 93:1102–15. 10.2522/ptj.20120454 23559522

[71] Hiengkaew V Jitaree K Chaiyawat P. Minimal detectable changes of the berg balance scale, fugl-meyer assessment scale, timed ‘Up & Go’ test, gait speeds, and 2-minute walk test in individuals with chronic stroke with different degrees of ankle plantarflexor tone. Arch Phys Med Rehabil. (2012) 93:1201–8. 10.1016/j.apmr.2012.01.014 22502805

[72] Mansfield A Wong JS Bryce J Knorr S Patterson KK. Does perturbation-based balance training prevent falls among individuals with chronic stroke? A randomised controlled trial. BMJ Open. (2018) 8:e021510. 10.1136/bmjopen-2018-021510 30121600 PMC6104758

[73] Mao H-F Hsueh I-P Tang P-F Sheu C-F Hsieh C-L. Analysis and comparison of the psychometric properties of three balance measures for stroke patients. Stroke. (2002) 33:1022–27. 10.1161/01.STR.0000012516.63191.C5 11935055

[74] Blum L Korner-Bitensky N. Usefulness of the berg balance scale in stroke rehabilitation: a systematic review. Phys Ther. (2008) 88:559–66. 10.2522/ptj.20070205 18292215

[75] Simpson LA Miller WC Eng JJ. Effect of stroke on fall rate, location and predictors: a prospective comparison of older adults with and without stroke. PLoS ONE. (2011) 6:e19431. 10.1371/journal.pone.0019431 21559367 PMC3084849

[76] El-Nashar H ElWishy A Helmy H El-Rwainy R Do core stability exercises improve upper limb function in chronic stroke patients? Egypt J Neurol Psychiatry Neurosurg. (2019) 55:38 10.1186/s41983-019-0087-6

[77] Van Criekinge T Saeys W Vereeck L De Hertogh W Truijen S. Are unstable support surfaces superior to stable support surfaces during trunk rehabilitation after stroke? A systematic review. Disabil Rehabil. (2018) 40:1981–8. 10.1080/09638288.2017.1323030 28482696

[78] Vera-Garcia FJ Grenier SG McGill SM. Abdominal muscle response during curl-ups on both stable and labile surfaces. Phys Ther. (2000) 80:564–9. 10.1093/ptj/80.6.564 10842409

[79] Kim J-H Kim Y Chung Y. The influence of an unstable surface on trunk and lower extremity muscle activities during variable bridging exercises. J Phys Ther Sci. (2014) 26:521–3. 10.1589/jpts.26.521 24764625 PMC3996413

[80] Chou S-W Wong AM Leong C-P Hong W-S Tang F-T Lin T-H. Postural control during sit-to stand and gait in stroke patients. Am J Phys Med Rehabil. (2003) 82:42–7. 10.1097/00002060-200301000-00007 12510184

[81] Duret C Grosmaire A-G Krebs HI. Robot-assisted therapy in upper extremity hemiparesis: overview of an evidence-based approach. Front Neurol. (2019) 10:412. 10.3389/fneur.2019.00412 31068898 PMC6491567

[82] Fazekas G Tavaszi I. The future role of robots in neuro-rehabilitation. Expert Rev Neurother. (2019) 19:471–3. 10.1080/14737175.2019.1617700 31090484

[83] Cirstea MC Levin MF. Compensatory strategies for reaching in stroke. Brain. (2000) 123:940–53. 10.1093/brain/123.5.940 10775539

[84] Levin MF Kleim JA Wolf SL. What do motor ‘recovery’ and ‘compensation’ mean in patients following stroke? Neurorehabil Neural Repair. (2008) 23:313–9. 10.1177/1545968308328727 19118128

[85] Garland SJ Willems DA Ivanova TD Miller KJ. Recovery of standing balance and functional mobility after stroke. Arch Phys Med Rehabil. (2003) 84:1753–9. 10.1016/j.apmr.2003.03.002 14669179

