# Supplementary material for: Dynamic Stability and Trunk Control Improvements Following Robotic Balance and Core Stability Training in Chronic Stroke Survivors: A Pilot Study
Source: Front Neurol. 2020 Jun 17;11:494. doi: 10.3389/fneur.2020.00494 (PMC7311757; doi:10.3389/fneur.2020.00494)
Supplement: Supplementary file 1 [file Data_Sheet_1.docx]

**Table S1. Steady state, proactive and reactive balance activities included in the training for the experimental and control group**

| ***Steady state* exercises** | | | | |
| --- | --- | --- | --- | --- |
|  | | | | |
| **Patient position: seated, with feet on the ground/ standing, with double leg base of support** | | | | |
| **Exercise: Balance on static seat/base** | | | | |
| **Task description:** the subjects is seated/standing on a static seat/base and has to maintain his load in the centre of the seat as much as possible, mantaining a correct postion of the trunk | | | | |
|  | **Modality of execution** | **Experimental condition** | **Difficulty regulation** | **Feedback** |
| *Control* | static base with therapis support | Open eyes  Closed eyes | -- | Verbal on postural allignment, based on observation |
| *Experimental* | *hunova* with static seat/base: the subject has to maintain the CoP in a defined area of confidence | Open eyes  Closed eyes | CoP Area of confidence | *Visual* (CoP position on the seat/base; trunk inclination on sagittal and frontal plane) based on seat force torque sensors and IMU sensor  *Auditory*, for high trunk oscillation based on IMU sensor |
| **Exercise: Balance on unstable seat/base** | | | | |
| **Task description:** the subjects is seated/standing on an unstable surface and has to maintain stability and balance as much as possible, mantaining a correct postion of the trunk | | | | |
|  | **Modality of execution** | **Experimental condition** | **Difficulty regulation** | **Feedback** |
| *Control* | proprioceptive wooden board/  Bobath ball | Open eyes  Closed eyes | -- | Verbal on postural allignment, based on observation |
| *Experimental* | *hunova* with unstable seat/base: the subject has to maintain the degree of seat oscillation in a defined area of confidence | Open eyes  Closed eyes | Seat/base maximum workspace  Seat/base oscillation area of confidence  Type of instability (proprioceptive, elastic or fluid dynamic):  Instability level | *Visual* (angular displacement of the seat/base; trunk inclination on sagittal and frontal plane) based on seat/base position sensors and IMU sensor  *Auditory*, for high trunk oscillation based on IMU sensor |

| ***Proactive* *balance* exercises** | | | | | |
| --- | --- | --- | --- | --- | --- |
|  | | | | | |
| **1) Patient position: seated, with feet on the ground** | | | | | |
| **Exercise: Pelvis mobilization** | | | | | |
| **Task description:** the subjects is seated on a moving/movable surface and movements for pelvis mobilization are performed | | | | | |
|  | **Modality of execution** | **Experimental condition** | | **Difficulty regulation** | **Feedback** |
| *Control* | Discosit chinesport/ Bobath ball  The patient performe active pelvis movements in the sagittal and frontal plane | Upper limbs in support or not | | -- | Verbal on postural allignment, based on observation |
| *Experimental* | *hunova* with moving seat (**passive mobilization**)  The seat moves the patient’s pelvis into a sagittal/frontal plane or following different trajectories  The patient has to stabilize the upper trunk maintaining the trunk inclination in a defined area of confidence | Upper limbs in support or not | | Amplitude of passive mobilization (degrees)  Velocity of movement  Trunk Area of confidence | Visual (trunk inclination on sagittal and frontal plane) based on IMU sensor  *Auditory*, for high trunk oscillation based on IMU sensor |
| *Experimental* | *hunova* with unstable seat (**active mobilization**)  the subject has to active move the seat for reaching targets on the screen in sagittal or frontal plane or in random directions. The exercise is associated with a gaming on the screen. |  | | Amplitude of active mobilization  Target positions | Visual (angular displacement of the seat; trunk inclination on sagittal and frontal plane) based on seat position sensors and IMU sensor  *Auditory*, for high trunk oscillation based on IMU sensor |
| **Exercise: Sit to stand** | | | | | |
| **Task description**: The patient has to stand up (sit to stand) | | | | | |
|  | **Modality of execution** | | **Experimental condition** | **Difficulty regulation** | **Feedback** |
| *Control* | static base with therapis support | |  |  | Verbal on postural allignment, based on observation |
| *Experimental* | *hunova* with static seat  The patient has to stand up maintaining the load distribution in a defined area of confidence | | Seat inclined of about 15°  Seat horizontal | Inclination of the seat  Load Area of confidence | *Visual* (load distribution during the sit to stand movement) based on base torque/force sensors |
|  | | | | | |
| **2) Patient position: seated, with feet on the ground or standing, with double leg base of support** | | | | | |
| **Exercise: Head and trunk rotation on static seat/base** | | | | | |
| **Task description:** the subject has to perform trunk or head rotations while maintaining a correct postural alignment and balance | | | | | |
|  | **Modality of execution** | **Experimental condition** | | **Difficulty regulation** | **Feedback** |
| *Control* | static base with therapis support | Open eyes  Closed eyes | | -- | Verbal on postural allignment, based on observation |
| *Experimental* | *hunova* with static seat/base  The subject has to maintain the CoP in a defined area of confidence | Open eyes  Closed eyes | | CoP Area of confidence | *Visual* (CoP position on the seat/base; trunk inclination on sagittal and frontal plane) based on seat/base force torque sensors and IMU sensor  *Auditory*, for high trunk oscillation based on IMU sensor |
| **Exercise: Motor dual task on static seat/base** | | | | | |
| **Task description**: the subjects has to perform movement with the upper libs while sitting on a static support | | | | | |
|  | **Modality of execution** | **Experimental condition** | | **Difficulty regulation** | **Feedback** |
| *Control* | static base with therapis support  The patient has to reach targets in the peri-personal space and extra-personal space. | -- | | -- | Verbal on postural allignment, based on observation |
| *Experimental* | *hunova* with static seat/base  The patient has to reach targets on the touch screen with the upper limbs. | -- | | -- | *Visual* (CoP position on the seat/base; trunk inclination on sagittal and frontal plane) based on seat/base force torque sensors and IMU sensor  *Auditory*, for high trunk oscillation based on IMU sensor |
|  | | | | | |
| **3) Patient position: standing, with double leg base of support** | | | | | |
| **Exercise: Limits of stability** | | | | | |
| **Task description:** the subjects stands on a moving/movable base and is requested to lean in different directions reaching his limits of stability | | | | | |
|  | **Modality of execution** | | **Experimental condition** | **Difficulty regulation** | **Feedback** |
| *Control* | Discosit chinesport with therapis support | | Open eyes  Closed eyes | -- | Verbal on postural allignment, based on observation |
| *Experimental* | *hunova* with passive moving base  The base moves the patient’s ankles into a sagittal/frontal plane or following different trajectories  The patient has to stabilize the upper trunk maintaining the trunk inclination in a defined area of confidence | | Open eyes  Closed eyes | Amplitude of passive mobilization (degrees)  Velocity of movement  Trunk Area of confidence | Visual (trunk inclination on sagittal and frontal plane) based on IMU sensor  *Auditory*, for high trunk oscillation based on IMU sensor |
| *Experimental* | *hunova* with unstable base  the subject has to mobilize the base for reaching targets on the screen in sagittal or frontal plane or in random directions. The exercise is associated with a gaming on the screen. | |  | Amplitude of active mobilization  Target positions | Visual (angular displacement of the platform; trunk inclination on sagittal and frontal plane) based on base position sensors and IMU sensor  *Auditory*, for high trunk oscillation based on IMU sensor |
|  | | | | | |
| **4) Patient position: standing, with asymmetric bipodalic base of support** | | | | | |
| **Exercise: Load bearing** | | | | | |
| **Task description**: the subjects has to shift the load chaniging from double leg base of support to one leg base of support | | | | | |
|  | **Modality of execution** | | **Experimental condition** | **Difficulty regulation** | **Feedback** |
| *Control* | Standing with ine leg on a static base/ Discosit chinesport /foam surface with therapis support  1) shifting the load to unimpaired /impaired leg  2) mainteining balance in asymettric bipodalic support with the unimpaired /impaired leg on a step  3) mainteining balance in asymettric bipodalic support with the unimpaired/impaired leg while reaching different positions with the unimpaired leg | | Step height and consistency  Footrest surface cosistency | Step heigh and consistency | Verbal on postural allignment, based on observation |
| *Experimental* | *hunova* with static/unstable base  1) the patient is in a bipodalic standing position and has to move toward a monopodalic condition on the unimpaired /impaired leg  2) mainteining balance in asymettric bipodalic support with the unimpaired /impaired on hunova and the unimpaired /impaired leg on a step  3) The patient is in a monopodalic standing position with the unimpaired/impaired leg on hunova and has to move the impaired leg for reaching different positions  the patient has to stabilize the upper trunk maintaining the trunk inclination in a defined area of confidence | | With static base  With unstable base | With static base:  CoP Area of confidence  Trunk Area of confidence  With unstable base:  Platform maximum workspace  Platform oscillation area of confidence  Type of instability (proprioceptive, elastic or fluid dynamic):  Instability level | *Visual* (CoP position on the base/ angular displacement of the base; trunk inclination on sagittal and frontal plane) based on base force torque/position sensors and IMU sensor  *Auditory*, for high trunk oscillation based on IMU sensor |

| ***Reactive balance* exercises** | | | | |
| --- | --- | --- | --- | --- |
|  | | | | |
| **Patient position: seated, with feet on the ground/ standing, with bipodalic base of support** | | | | |
| **Exercise: Response to perturbations – Reative balance** | | | | |
| **Task description:** the subjects has to maintain balance reacting to perturbations | | | | |
|  | **Modality of execution** | **Experimental condition** | **Difficulty regulation** | **Feedback** |
| *Control* | Bobath ball/ Discosit chinesport  The therapist induces perturbations to the subject | Upper limbs in support or not | --- | Verbal on postural allignment, based on observation |
| *Experimental* | *hunova* with passive moving seat/base  The seat/base moves inducing random perturbations  The patient has to stabilize the upper trunk maintaining the trunk inclination in a defined area of confidence | Upper limbs in support or not | Amplitude of perturbations (degrees)  Velocity of perturbations  Trunk Area of confidence | Visual (trunk inclination on sagittal and frontal plane) based on IMU sensor  *Auditory*, for high trunk oscillation based on IMU sensor |
|  | | | | |
| **Exercise: Motor dual task on unstable seat/base** | | | | |
| **Task description:** the subjects has to perform movement with the upper limbs while sitting on a dynamic support | | | | |
|  | **Modality of execution** | **Experimental condition** | **Difficulty regulation** | **Feedback** |
| *Control* | Bobath ball/ Discosit chinesport  The patient has to reach targets in the peri-personal space and extra-personal space. | Upper limbs in support or not | -- | Verbal on postural allignment, based on observation |
| *Experimental* | *hunova* with unstable seat/base  The subject has to maintain the degree of seat oscillation in a defined area of confidence while reaching targets on the touch screen with the upper limbs. | Upper limbs in support or not | Seat/base maximum workspace  Seat/base oscillation area of confidence  Type of instability (proprioceptive, elastic or fluid dynamic):  Instability level | *Visual* (angular displacement of the seat/base; trunk inclination on sagittal and frontal plane) based on seat position sensors and IMU sensor  *Auditory*, for high trunk oscillation based on IMU sensor |

|  |  | **Berg Balance Scale (BBS)** | | | **MiniBEST Test (MBT)** | | | **Trunk Impairment Scale (TIS)** | | |
| --- | --- | --- | --- | --- | --- | --- | --- | --- | --- | --- |
|  | **ID** | **T0** | **T1-T0**  **(% change T0-T1)** | **T2-T0**  **(% change T0-T2)** | **T0** | **T1-T0**  **(% change T0-T1)** | **T2-T0**  **(% change T0-T2)** | **T0** | **T1-T0**  **(% change T0-T1)** | **T2-T0**  **(% change**  **T0-T2)** |
| **EXPERIMENTAL** | **1** | 49 | 0 (0%) | 2 (4.08%) | 15 | 2(13.33%) | -1(-6.66%) | 13 | -1(-7.69%) | 1(7.69%) |
|  | **2** | 50 | 0 (0%) | 6 (12%) | 17 | 2(11.76%) | 2(11.76%) | 14 | 0(0%) | 0(0%) |
|  | **6** | 47 | 9 (19.14%) | 4 (8.51%) | 17 | 4(23.52%) | 0(0%) | 13 | 5(38.46%) | 2(15.38%) |
|  | **9** | 45 | 6 (13.3%) | 2 (4.44%) | 11 | 5(45.45%) | 5(45.45%) | 10 | 5(50%) | 4(40%) |
|  | **11** | 47 | 4 (8.51%) | 1 (2.12%) | 16 | 2(12.5%) | -1(-6.25%) | 14 | 2(14.28%) | -1(-7.14%) |
|  | **13** | 46 | 3 (6.52%) | 0 (0%) | 9 | 11(122.22%) | 5(55.55%) | 12 | 4(33.33%) | 1(8.33%) |
|  | **15** | 47 | 9 (19.14%) | 8 (17.02%) | 21 | 1(4.76%) | 2(9.52%) | 12 | 2(16.66%) | -1(-8.33%) |
|  | **17** | 41 | 4 (9.75%) | 6 (14.63%) | 15 | 0(0%) | 2(13.33%) | 9 | 3(33.33%) | 2(22.22%) |
|  | **21** | 48 | 0 (0%) | 0 (0%) | 18 | 1(5.55%) | 1(5.55%) | 12 | 3(25%) | 0(0%) |
|  | **22** | 54 | 1 (1.85%) | 1 (1.85%) | 21 | 1(4.76%) | 1(4.76%) | 13 | 0(0%) | 2(15.38%) |
|  | **23** | 49 | 0 (0%) | 1 (2.04%) | 11 | 3(27.27%) | 3(27.27%) | 12 | 0(0%) | 1(8.33%) |
|  | **25** | 56 | 0 (0%) | 0 (0%) | 24 | 0(0%) | 1(4.16%) | 15 | 0(0%) | 6(40%) |
|  | **29** | 51 | 1 (1.96%) | 4 (7.84%) | 21 | 0(0%) | 0(0%) | 16 | -1(-6.25%) | 0(0%) |
|  | **30** | 44 | 1 (2.27%) | 0 (0%) | 11 | 6(60%) | 0(0%) | 11 | 5(45.45%) | -1(-9.09%) |
|  |  |  |  |  |  |  |  |  |  |  |
| **CONTROL** | **3** | 54 | 1 (1.85%) | 2 (3.70%) | 16 | 3(18.75%) | 5(31.25%) | 14 | 3(21.42%) | 3(21.42%) |
|  | **4** | 54 | 0 (0%) | 2 (3.70%) | 21 | 1(4.76%) | 2(9.52%) | 13 | 0(0%) | 0(0%) |
|  | **5** | 51 | 5 (9.80%) | 5 (9.80) | 22 | 2(9.09%) | 2(9.09%) | 13 | 1(7.69%) | 2(15.38%) |
|  | **7** | 50 | 3 (6%) | 3 (6%) | 16 | 5(31.25%) | 5(31.25%) | 14 | 7(50%) | 2(14.28%) |
|  | **12** | 46 | -5 (-10.86%) | -5 (-10.86%) | 13 | 0(0%) | -5(-38.46%) | 13 | 1(7.69%) | -1(-7.69%) |
|  | **14** | 53 | 1 (1.88%) | -4 (-7.54%) | 19 | 5(26.31%) | 0(0%) | 12 | 4(33.33%) | 3(25%) |
|  | **16** | 50 | 6 (12%) | 4 (8%) | 21 | 1(4.76%) | 1(4.76%) | 12 | 3(25%) | 0(0%) |
|  | **18** | 41 | 0 (0%) | 0 (0%) | 2 | 0(0%) | 2(100%) | 10 | 0(0%) | 0(0%) |
|  | **19** | 42 | 0 (0%) | 1 (2.38%) | 10 | 3(30%) | 2(20%) | 13 | 1(7.69%) | -2(15.38%) |
|  | **20** | 51 | 1 (1.96%) | 2 (3.92%) | 18 | 1(5.55%) | 3(16.66%) | 13 | 2(15.38%) | 0(0%) |
|  | **24** | 56 | 0 (0%) | 0 (0%) | 23 | 0(0%) | -3(-13.04%) | 15 | -1(-6.66%) | -2(-13.33%) |
|  | **27** | 41 | 1 (2.43%) | 2 (4.87%) | 8 | 0(0%) | 1(12.5%) | 4 | 6(150%) | 1(25%) |
|  | **28** | 52 | 3 (5.76%) | 3 (5.76%) | 21 | 1(4.76%) | -1(-4.76%) | 13 | 1(7.69%) | -1(-7.69%) |

**Table S2.** Invidual subjects improvements for BBS, MiniBEST Test and Trunk Impairment scales. For each subjects are reported baseline values (T0), improvement at T1 (ad the relative % of imporvement with respect to T0) and the imporvement at T2 (ad the relative % of imporvement with respect to T0

|  | **Direction of perturbation** | | | |
| --- | --- | --- | --- | --- |
| **Performance at T1** | **Forward**  **(# of subjects)** | **Backward**  **(# of subjects)** | **Affected side**  **(# of subjects)** | **Not-affected side**  **(# of subjects)** |
| **Stable at 8 degrees** | 18 (8E-10C) | 15 (7E-8C) | 19 (9E-10C) | 17 (10E-7C) |
| **Improved** | 5 (4E-1C) | 7 (5E-2C) | 5 (3E-2C) | 7 (2E-5C) |
| **Decreased** |  | 1 (1C) | 1 (1C) |  |
| **Stable below 8 degrees** | 2 (2C) | 2 (2C) |  | 1 (1C) |
| **Test not performed** | 2 (1E-1C) | 2 (1E-1C) | 2 (1E-1C) | 2 (1E-1C) |

**Table S3.** Reactive balance test performance at T1 with respect to T0. E= experimental group; C=control group.
